# A record thermoelectric efficiency in tellurium-free modules for low-grade waste heat recovery

**DOI:** 10.1038/s41467-021-27916-y

**Published:** 2022-01-11

**Authors:** Zhonglin Bu, Xinyue Zhang, Yixin Hu, Zhiwei Chen, Siqi Lin, Wen Li, Chong Xiao, Yanzhong Pei

**Affiliations:** 1 Interdisciplinary Materials Research Center, School of Materials Science and Engineering, Tongji Univ., 4800 Caoan Rd., Shanghai, 201804 China; 2 Hefei National Laboratory for Physical Sciences at the Microscale, University of Science and Technology of China, Hefei, Anhui 230026 P. R. China

**Keywords:** Thermoelectrics, Thermoelectric devices and materials

## Abstract

Low-grade heat accounts for >50% of the total dissipated heat sources in industries. An efficient recovery of low-grade heat into useful electricity not only reduces the consumption of fossil-fuels but also releases the subsequential environmental-crisis. Thermoelectricity offers an ideal solution, yet low-temperature efficient materials have continuously been limited to Bi_2_Te_3_-alloys since the discovery in 1950s. Scarcity of tellurium and the strong property anisotropy cause high-cost in both raw-materials and synthesis/processing. Here we demonstrate cheap polycrystalline antimonides for even more efficient thermoelectric waste-heat recovery within 600 K than conventional tellurides. This is enabled by a design of Ni/Fe/Mg_3_SbBi and Ni/Sb/CdSb contacts for both a prevention of chemical diffusion and a low interfacial resistivity, realizing a record and stable module efficiency at a temperature difference of 270 K. In addition, the raw-material cost  to the output power ratio in this work is reduced to be only 1/15 of that of conventional Bi_2_Te_3_-modules.

## Abstract (web summary)

Thermoelectric materials for low-grade heat recovery applications are limited to Bi_2_Te_3_-based alloys containing expensive Te for decades. Here, the authors demonstrate on a module level, cheap antimonides could enable an efficiency not inferior to that of expensive tellurides.

## Introduction

**Fig. 1:**
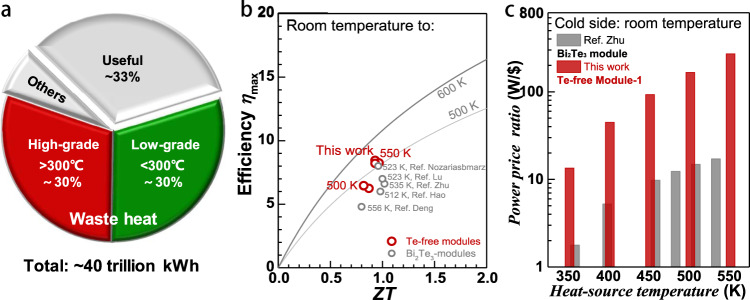
Energy consumption distribution and thermoelectric performance. Energy consumption distribution indicating the huge proportion of low-grade waste heat (*T* < 300 °C)^7,47^ (**a**), device *ZT* dependent maximum efficiency (*η*_max_)^5,18,48,49^ (**b**) and heat-source temperature-dependent power price ratio (W/$)^18^ at a conventional leg size of 1.5 × 1.5 × 2 mm^3^ for Module–1 (**c**).

Modern industries intensively consume energy. In various manufacturing and process plants, over 60% of total energy is dissipated as waste heat^1,2^. The high-temperature high-quality waste heat can easily be collected and re-used in industries. However, it is challenging to harvest and re-use the low-temperature waste heat (mostly emitted in the range of <300 °C^3–5^) through conventional energy conversion technologies because it is generally of low quality and low energy density^5^. This low-grade waste heat actually accounts for 50% of the total (Fig. 1a)^6,7^. As a clean and silent energy conversion technology, thermoelectric power generation enables a direct conversion of waste heat into useful electricity, which reduces fossil fuel consumption and releases the subsequential environmental crisis.

The conversion efficiency of a thermoelectric device mainly depends on the materials’ figure of merit *zT* = *S*^2^*T*/*ρκ*, where *S*, *T, ρ, κ* are Seebeck coefficient, absolute temperature, electrical conductivity and thermal conductivity, respectively. Obviously, this indicates the greater challenge for realizing a high *zT* at low temperatures, and indeed *zT* at mid-to-high temperatures are significantly improved in materials including PbTe^8,9^, Half-heusler^10,11^, SiGe^12,13^, and GeTe^14–16^. In the temperature range of low-grade waste heat (<600 K), high-performance thermoelectric materials have long been limited to Bi_2_Te_3_-alloys^5,17^. In fact, Bi_2_Te_3_-based thermoelectrics in both n- and p-type are so far the only materials being commercialized for recovering low-grade waste heat and refrigeration applications^5,18,19^.

Although Bi_2_Te_3_-alloys are thermodynamically stable at higher temperatures, the thermoelectric devices are limited to be properly operated below 450 K, because of the strong detrimental bipolar effect^5,19,20^ induced by the narrow bandgap at *T* > 450 K. This results in a typical efficiency of only ~7% or less for available low-grade waste heat recovery devices (Fig. 1b)^5,18^. In addition, the scarcity of tellurium leads the cost and sustainability to be continuous concerns of conventional Bi_2_Te_3_ thermoelectrics. The maximum output power to the raw-material cost ratio for Bi_2_Te_3_ thermoelectrics is about 17 W/$ (power price ratio, Fig. 1c)^18^. Moreover, the high thermoelectric performance of Bi_2_Te_3_-alloys is limited to along particular crystallographic directions at an expense of poor machinability due to the strongly anisotropic crystal structure, which further raises the device fabrication cost. Therefore, seeking cheap and high-efficiency alternatives for low-grade waste heat recovery (*T* < 600 K) has long been an anticipation in this field.

A construction of a thermoelectric device generally required both n- and p-type materials with similarly high performance. Recently, cheap n-type Mg_3_Sb_2_^21–25^ was reported not only to enable a better refrigeration effect as the n-leg of a device replacing n-type Bi_2_Te_3_^26^, but also to show a high single-leg efficiency of ~10% for power generation at a source temperature of *T* ~ 700 K^27–29^. Furthermore, a uni-couple device of n-Mg_3_Sb_2_ and p-Bi_2_Te_3_ materials show a high conversion efficiency of 9% using a source temperature of ~573 K^30^. Things do not go so well for seeking p-type alternatives to Bi_2_Te_3_. Although p-type MgAgSb^31,32^ was reported to have a *zT* comparable to p-type Bi_2_Te_3_, and a single-leg device efficiency of ~8% at a source temperature of *T* ~ 550 K^33^, phase transitions in this compound with expensive Ag might bring extra challenges for mass production^33^. Fortunately, p-type CdSb^34,35^ was reported to show a high average *zT* > 1 at *T* < 600 K, which is comparable to p-type Bi_2_Te_3_. Therefore, a device assembled by cheap materials of n-type Mg_3_Sb_2_ and p-type CdSb is expected to be a promising solution for efficient low-grade waste heat recovery without using Bi_2_Te_3_-alloys.

**Fig. 2:**
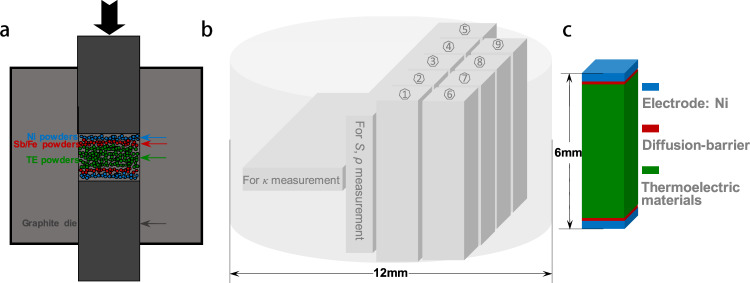
Schematic of the tellurium-free legs fabricated. Schematic of the powder packing of the one-step hot-pressing (**a**), schematic slicing diagram of the cylinders (**b**), and schematic of a leg showing the contact structure (**c**).

Here, we focus on both material- and device-level properties of n-Mg_3.1_Y_0.01_BiSb and p-Cd_0.99_Ag_0.01_Sb, both of which consist of abundant elements and are optimized to show an average *zT* (*zT*_avg_) of >1 within ambient temperature to 600 K. In addition to a high material’s *zT*, an electrically and thermally conductive but chemically inert contacts between thermoelectric materials and electrodes are essential to ensure a high device efficiency. Usually, a stack of multiple layers can be applied for a prevention of atomic diffusion, a release of thermal stress, and a reduction of contact resistance. These are enabled in this work by a one-step hot-pressing process for large cylinders of n-type and p-type containing Ni electrodes and diffusion barrier layers (Fe for Mg_3.1_Y_0.01_BiSb and Sb for CdSb, Fig. 2). The tellurium-free thermoelectric modules with a very high power-price ratio of 272 W/$ was assembled to show a high efficiency more than 8% at *T* ≤ 550 K, using legs sliced from the hot-pressed cylinders. This offers great potentials of the tellurium-free thermoelectrics here as economic, sustainable, and efficient alternatives to conventional Bi_2_Te_3_-based ones for low-grade waste heat recovery.

## Results

### Tellurium-free legs fabricated and materials properties

The details on material synthesis, module fabrication (dimensions shown in Supplementary Table 1), characterizations, and property measurements (efficiency measurement setup in Supplementary Fig. 1) are given in the Supplementary. A copper bar with known thermal conductivity was used as a heat-flow meter within a temperature difference of 0.6–2.3 K, which is determined by averaging 60 measurements with a relative standard deviation of <3%^36^ (Supplementary Table 2, Supplementary Fig. 2). Details on the estimated horizontal and vertical heat radiations, the response of the heat-flow meter and the stable open-circuit thermoelectromotive force are shown in Supplementary Fig. 3–6 and Supplementary Table 3. Details on calibrating the measurement system are shown in Supplementary Fig. 7–10, with comparisons to the results measured by commercial instruments from both the material and device levels. The hot-pressed cylinders are sliced along (for *κ* measurement) and perpendicular (for *S*, *ρ* measurement) to the pressure direction (Fig. 2b). The X-ray diffraction (XRD) patterns, scanning electron microscopy (SEM) images and corresponding energy dispersive spectroscopy (EDS) compositional mapping for n-type Mg_3_SbBi and p-type CdSb are shown in Supplementary Fig. 11. No obvious impurity peaks were observed from XRD patterns for both materials, indicating a good phase purity and SEM and EDS results further confirm a good homogeneity.

**Fig. 3:**
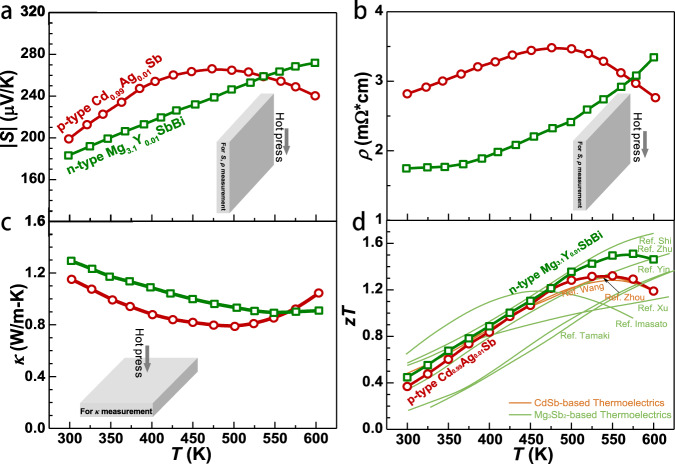
Temperature dependent transport properties. Temperature-dependent Seebeck coefficient (*S*) (**a**), electrical resistivity (*ρ*) (**b**), thermal conductivity (*κ*) (**c**), and thermoelectric figure of merit (*zT*) (**d**) for n-type Mg_3.1_Y_0.01_SbBi and p-type Cd_0.99_Ag_0.01_Sb with a comparison to literature results^21,22,25,27,34,35,37,38^, where the inset schematically shows the electronic and thermal transport properties measured along the hot-press direction.

Temperature-dependent electronic and thermal transport properties of both *n*-Mg_3.1_Y_0.01_BiSb and *p*-Cd_0.99_Ag_0.01_Sb, all measured along the hot-press direction, are shown in Fig. 3. This work obtains comparable thermoelectric performances to the literature results (Fig. 3d)^21,22,27,34,35,37,38^. Although the *zT* of both materials here are inferior to that of Bi_2_Te_3_ near room temperature, the much higher *zT* at 450–600 K ensures a high average *zT*, leading these materials to be reasonable choices for power generation within 300–600 K (Supplementary Fig. 12, 13). This can be understood by the strong detrimental bipolar effect at *T* > 450 K^5,19,20^, induced by the narrow bandgap in Bi_2_Te_3_-thermoelectrics.

The stability of the thermoelectric materials is primary for a stable module. Thermoelectric CdSb and ZnSb have been known since 1950s^39,40^, yet never been assembled for a module. In this work, the safe operating temperature of CdSb was determined to be ~550 K or below. In more details, XRD patterns for both ingot quenched form melt and annealed samples are given in Supplementary Fig. 14. Ingot quenched from melt actually contains Cd_13_Sb_10_ as the matrix with elemental Sb as an impurity phase. While the post-annealing at 673 K for 48-hours enables a homogenous crystallization of CdSb phase. This is very consistent with the literature work^35^. In addition, the SEM images and the EDS mapping confirm a complete formation of CdSb phase within the temperature range focused on in this work (Supplementary Fig. 14). The electronic properties of two different CdSb samples aging at 550 K become much more stable, according to the ~500 measurements in about 250 h for each sample (Supplementary Fig. 15). Similarly, the properties of the mating n-type leg of Mg_3_SbBi are quite stable as well at an even higher aging temperature of 600 K. Moreover, good stability in thermal conductivity is confirmed by the measurements before and after aging (Supplementary Fig. 16).

### Module optimization and performances

To maximize the module efficiency, the ratio of cross-sectional areas of p- and n-type legs needs to be optimized to ensure both high performances of legs at the same current density^41^. This requires a geometric configuration of n- and p-type legs that satisfies the relation of (*L*_n_*A*_p_)/(*L*_p_*A*_n_) = (*K*_n_*R*_p_/*K*_p_*R*_n_)^0.5 41^. Here, *L*, *A*, *K* and *R* are the length, cross-sectional area, thermal conductance (*K* = *κA*/*L*) and electrical resistance (*R* = *ρL*/*A*), and superscripts n and p indicate the n- and p-type legs, respectively. Due to the similar Seebeck coefficient, electrical resistivity and thermal conductivity in both *n*-type Mg_3_SbBi and *p*-type CdSb within 300–550 K, *A*_n_/*A*_p_ = 1 is eventually chosen to fabricate three Mg_3_SbBi/CdSb thermoelectric modules with a leg size of 1.5 × 1.5 mm^2^ in cross section. To establish a reliable temperature difference across the thermoelectric legs, the materials were chosen in length of 2 mm (for Module-1) or 4 mm (for Module-2 and 3), which also can maintain a reasonably small contribution (<5%) of contact resistance to the internal resistance (Supplementary Fig. 17).

**Fig. 4:**
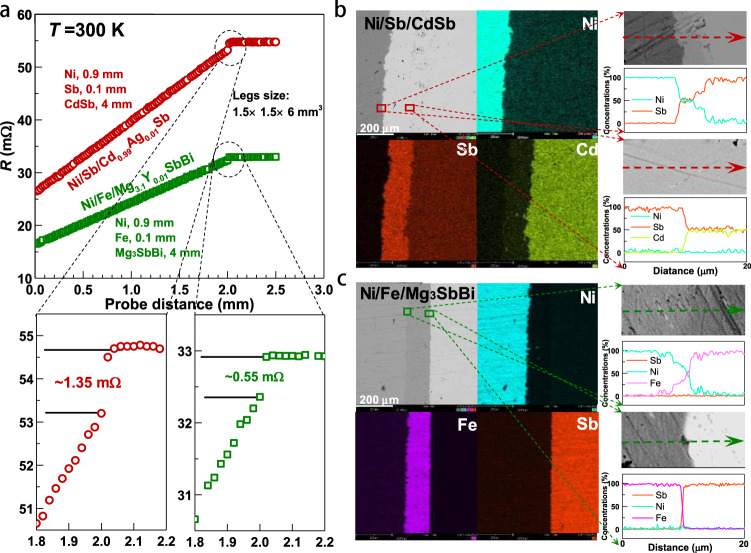
Contact structure and resistance. Line scanning of resistance (*R*) across the Ni/Sb/CdSb and Ni/Fe/Mg_3_SbBi interfaces for estimating the contact resistance (**a**), SEM images, EDS mapping, and EDS line scanning for the Ni/Sb/CdSb (**b**) and the Ni/Fe/Mg_3_SbBi junctions (**c**).

For a device, contact resistance needs to be minimized to ensure a low device internal resistance (*R*_in_) thus a maximum output power (*P*_max_) and efficiency (*η*). The electrical contact resistance (*R*_c_) was estimated according to a linear resistance scanning measurement based on a four-probe technique^42^. Multiplying the contact resistance (*R*_c_) with the contact area, the interfacial contact resistivity (*ρ*_c_) can be obtained^43^. In order to obtain a low contact resistance, as well as a robust bonding, various contact designs were designed (Supplementary Fig. 18, Supplementary Table 4). Among these metals, the contact resistivity of Ni/CdSb structure is the lowest one. However, the Ni can easily react with CdSb, leading to a significant aggregation of Sb at the boundary (Supplementary Fig. 19). This hints excess of Sb at the boundary might be helpful for reducing the diffusion. According to previous works showing a very low contact resistivity^6,44^, native elements or inter-compounds of the thermoelectric materials themselves seem to work well as efficient diffusion barrier layers with low interfacial contact resistivity. A sandwich structure of Ni/Sb/CdSb (Ni as the electrode and Sb as the diffusion barrier layer) is chosen in this work since it enables the lowest interfacial contact resistivity (*ρ*_c_) of only ~30 μΩ cm^2^ (Fig. 4, Supplementary Table 4). Previous literature reports a low *ρ*_c_ in Fe/Mg_3_Sb_2_^28^. In this work, Fe and Ni were respectively used as the diffusion barrier and electrode of the n-type Mg_3_SbBi legs, and the obtained *ρ*_c_ of ~12 μΩ cm^2^ is comparable to the literature results^28^. The small difference of interfacial contact resistance between different legs indicates the rationality of the structure here in both Ni/Sb/CdSb and Ni/Fe/Mg_3_SbBi (Supplementary Fig. 20). Furthermore, the linearly increasing resistance with distance in the CdSb and Mg_3_SbBi layers indicate that the electrical conductivity of thermoelectric materials are almost constant throughout the scanned area, confirming the high homogeneity. In addition, SEM observation and EDS mapping indicate good bonding with negligible chemical diffusion in both p-type CdSb legs and n-type Mg_3_SbBi legs, suggesting the rationality of choices of diffusion barrier materials here (Fig. 4b, c).

**Fig. 5:**
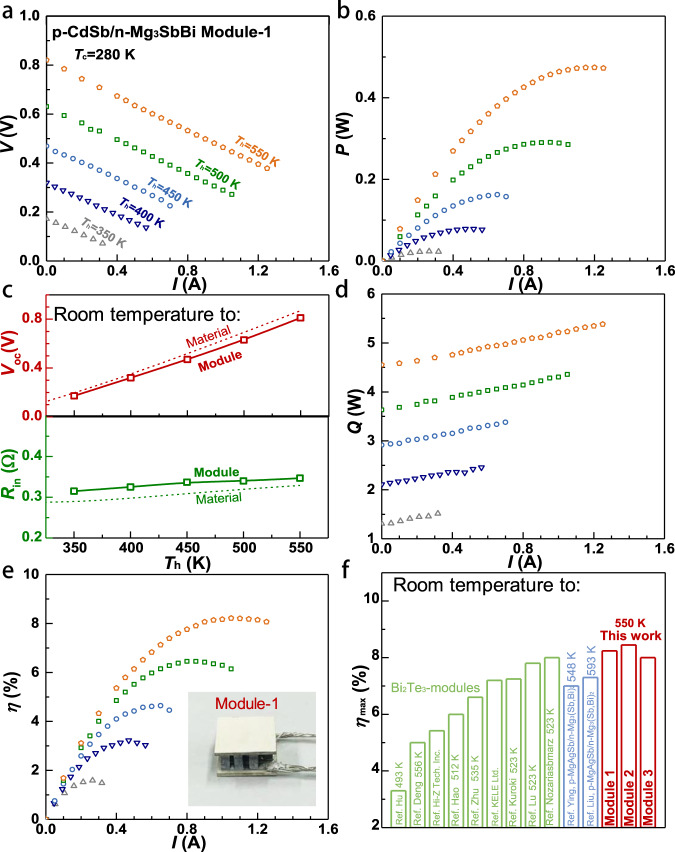
Module properties. Current (*I*) dependent output voltage (*V*) (**a**) and output power (*P*) (**b**), open-circuit voltage (*V*_oc_) and internal resistance (*R*_in_) (**c**), heat-flow (*Q*) from cold-side (**d**), conversion efficiency (*η*) (**e**) and its maximum *η*_max_ (**f**) under hot-side temperature of 550 K for CdSb/Mg_3_SbBi-modules. Literature results of Bi_2_Te_3_-modules and MgAgSb/Mg_3_SbBi-modules are included for a comparison^5,6,18,48–55^.

The device properties for modules are shown in Fig. 5, Supplementary Fig. 21 and 22. The nice linearity of current (*I*) versus output voltage (*V*) suggests a nearly Ohmic device in this work. The intercept and slope represent the open-circuit voltage (*V*_oc_) and the module’s internal resistance (*R*_in_), respectively. For comparisons, *R*_in_ and *V*_oc_ are also estimated according to the material’s properties. The discrepancy between the estimated and measured *R*_in_ can be ascribed to the contact resistance. The good agreement between measurement and estimation in *V*_oc_ of all modules suggests a negligible temperature difference loss at the contacts even though the thermoelectric materials vary in length. The increase in *R*_in_ and *V*_oc_ with increasing temperature difference can be respectively understood by the increase at high temperatures in resistivity and Seebeck coefficient of both Cd_0.99_Ag_0.01_Sb and Mg_3.1_Y_0.01_BiSb.

The output power (*P*) first increases with increasing current, reaches its maximum when the external load resistance matches with *R*_in_, and then decreases. The maximum output power is ~0.474 W at the working condition of *T*_h_ = 550 K and Δ*T* = 270 K of Module-1, corresponding to a power density (*P*_d_, P over the total sectional area of legs) as high as 15 kW/m^2^. An exact fair comparison on output power (*P*) or output power density (*P*_d_) of different modules having different heights are difficult, because of the existence of contact resistance. To make the comparison more straightforward, Module-1 is fabricated with thermoelectric legs of 2 mm tall, which is very comparable in size to conventional Bi_2_Te_3_-modules^5,18^. In principle, specific maximum power density (*P*_d_ × *L*, with *L* as the leg length)^45^, offers an advantage over *P*_d_, because it becomes length *L* independent in case of zero contact resistance. *R*_in_ increases with increasing *L* (Supplementary Fig. 17), therefore, *P*_d_ is *L*-dependent. However, the product *P*_d_ × *L* roughly remains unchanged, because the contact resistance (*R*_c_) is overall small as compared to *R*_in_. The contribution of *R*_c_ to *R*_in_ can be reduced by increasing *L*, leading *P*_d_ × *L* to show a saturation as *L* is large, rationalizing a comparison on *P*_d_ × *L* between modules consisting of different thermoelectric materials^46^. Indeed, both *P*_d_ and *P*_d_ × *L* of Module-1 are comparable to those of Bi_2_Te_3_ modules^5,18^ with similar dimensions (Supplementary Fig. 23).

A maximum and stable high efficiency (*η*_max_) of >8% is realized at a hot-side temperature of 550 K and cold-side temperature of 280 K (Fig. 5, Supplementary Fig. 21). This efficiency is actually higher than any of the experimental results in various modules ever reported, which can be understood by the high *zT* as well as the stable compatibility factor of these antimonides at these temperatures (Supplementary Fig. 12 and Fig. 24). Note the optimal currents for maximizing output power *P*_max_ and efficiency *η*_max_ are slightly different due to the Joule heating and Peltier effects that would increase the total heat flow (Fig. 5d). The measured heat flow *(Q)* at conditions of open circuit (*η* = 0) and of maximum efficiency (*η* = *η*_max_) are quite comparable to the predictions (Supplementary Fig. 24a), and the discrepancy at high-temperature gradients can be ascribed to the vertical heat radiation from the unfilled hot-side to the cold-side substrate (Supplementary Fig. 4 and Fig. 5). Similarly, the measured *η*_max_ reasonably agrees with the prediction (Supplementary Fig. 24), indicating a nearly realization of the full potential of high-performance CdSb/Mg_3_SbBi thermoelectric materials here. Note that a shorter leg length in Module-1 corresponds to a higher contact resistance ratio, thus a lower specific power density (*P*_d_ × *L*) and a lower efficiency (*η*_max_) as compared to that in Module-2 with a longer leg length. Overall, the small deviation in measured and predicted *η*_max_ further suggests the rational design of the module in this work. Both horizontal and vertical heat radiations are taken into account here for estimating the efficiency, and the corresponding maximum efficiency are listed in Supplementary Table 3 for Module-1.

In addition to the high efficiency, the module in this work shows a very good stability, which is evidenced by continuous output power measurements under 555 K for 200 h (Supplementary Fig. 25). Due to the high stability of the thermoelectric materials at the safe operating temperature of < ~550 K, a continuous operating under this temperature for ~200 h leads the module efficiency to decrease by only <3%, which actually falls in the measurement uncertainty range (Supplementary Fig. 25). Furthermore, the stability of CdSb/Mg_3_SbBi module in this work is ensured by cycling test with a hot-side temperature of 555 K. In more details, the hot-side temperature of Module-1 ramps up from 350 K to 550 K for 15 times at a different constant output current for each time. Such a cycling test enables a maximum efficiency of ~8% when the output current is about 0.8-1.2 A (Supplementary Fig. 26 and Fig. 27). This module efficiency is highly comparable to that of measured before and after the 15-times thermal cycling using a steady-temperature-gradient technique, which further confirms the excellent responsivity of the measurement setup. All these results ensured a stable module efficiency of >8% at an operating hot-side temperature of 555 K has high thermal cycle stability. Furthermore, the interfacial contact resistance for the p-type CdSb leg increases form an initial 1.4 mΩ to 2.1 mΩ and then to 2.7 mΩ, due to a 14-days and an 80-days aging, respectively (Supplementary Fig. 20 and Fig. 28). Correspondingly for the n-type Mg_3_SbBi leg, contact resistance increases form an initial 0.6 mΩ to 1.7 mΩ and then to 2.4 mΩ. It is the overall small contact resistivity of <60 μΩ cm^2^ guaranteeing the excellent long-term service stability (Supplementary Fig. 25 and Fig. 26). In addition, SEM observations taken after an eighty-day aging at 550 K consistently confirm the robust contacts with clear boundaries (Supplementary Fig. 29). Along with the detailed continuous measurements of electronic properties during aging/cycling, one could conclude that the materials and modules in this work show a very good stability.

This indicates the excellent chemical, thermal, electrical, and mechanical stability of the contacts in this work. Last but important, the abundance and yield of all constituent elements for Mg_3_SbBi/CdSb module here are much larger than that in Bi_2_Te_3_-based ones, leading the raw-material cost of the Mg_3_SbBi/CdSb module is only one-tenth of that of conventional Bi_2_Te_3_-based modules (Supplementary Table 5). At a conventionally leg size of 1.5× 1.5× 2 mm^3^, the power price ratio of 272 W/$ realized in the antimonide module here is much higher than that of Bi_2_Te_3_-based one (17 W/$)^18^. Note the lower density of these antimonides offers an extra advantage for the power price ratio.

## Discussion

In summary, a high thermoelectric module efficiency of 8% at a heat-source temperature of *T* < 550 K is realized using tellurium-free materials. This is enabled by the high thermoelectric performance of both n- and p-type materials as well as an effective interfacial contact design, the latter of which ensures a low contact resistance and a prevention of chemical diffusion. The constituent elements of these antimonides here are much cheaper and abundant as compared to those of conventional Bi_2_Te_3_-based modules. This work offers reasonable opportunities to challenge the long-term monopoly of Bi_2_Te_3_-based modules for efficient low-grade waste heat recovery.

## Methods

### Synthesis

Polycrystalline p-type Cd_0.99_Ag_0.01_Sb was synthesized by melting stoichiometric amount of high purity elements Cd (99.999%), Ag (99.999%), Sb (99.99%) at 873 K for 5 h, followed by quenching in cold water and annealing at 673 K for 48 h. The obtained Cd_0.99_Ag_0.01_Sb ingot was ground into fine powder for further hot pressing. The Cd_0.99_Ag_0.01_Sb powders, Sb powders and Ni powders were loaded to a graphite die for one-step hot pressing at 658 K for 90 min under a uniaxial pressure of ~65 MPa, obtaining a Ni/Sb/Cd_0.99_Ag_0.01_Sb/Sb/Ni cylinder with dimensions of ~12 mm in diameter and ~3 or 6 mm in thickness (Ni layer: ~0.4 or 0.9 mm, Sb layer: ~0.1 mm, Cd_0.99_Ag_0.01_Sb layer: ~2 or 4 mm). The Sb, Fe, and Ni powders used as diffusion layers or electrodes cam with a particle size of 200 mesh.

Polycrystalline n-type Mg_3.1_Y_0.01_SbBi was synthesized by melting stoichiometric amount of high purity elements Mg (99.99%), Y (99.99%), Sb (99.99%), Bi (99.99%) in a sealed tantalum tube in a quartz ampoule at 1323 K for 5 h, followed by quenching in cold water and annealing at 923 K for 48 h. The sealing of the tantalum tube was done with an arc-melting system in argon with a pressure slightly lower than the atmosphere. Then the obtained Mg_3.1_Y_0.01_SbBi ingot was ground into powder. The Mg_3.1_Y_0.01_SbBi powders, Fe powders and Ni powders were loaded into a graphite die for one-step hot pressing at 823 K for 90 min under a uniaxial pressure of ~90 MPa, obtaining a Ni/Fe/Mg_3.1_Y_0.01_SbBi/Fe/Ni cylinder with dimensions of ~12 mm in diameter and ~3 or 6 mm in thickness (Ni layer: ~0.4 or 0.9 mm, Fe layer: ~0.1 mm, Mg_3.1_Y_0.01_SbBi layer: ~2 or 4 mm).

The obtained n- and p-type cylinders were then sliced into plates and bars using a Wire-Cutting-Machine for characterizations, transport property measurements and module fabrication (Fig. 2).

### Characterizations and transport property measurements

Phase compositions of both p-Cd_0.99_Ag_0.01_Sb and n-Mg_3.1_Y_0.01_SbBi samples were characterized by X-Ray diffraction (XRD, DX2000). Both thermoelectric materials and junctions were characterized by Scanning Electronic Microscopy (SEM, Phenom Pro) equipped with an Energy Dispersive Spectrometer (EDS). Resistivity and Seebeck coefficient were simultaneously measured at various temperatures under helium. A four-probe Van Der Pauw technique was used for the resistivity measurement. The Seebeck coefficient was determined by the slope of thermopower versus temperature difference within 0–5 K recorded by two K-type thermocouples attached to the edges of a radial direction of the sample. The thermal conductivity (*к*) is estimated by *к = dC*_p_*D*, where *d* is the density, *C*_p_ is the heat capacity of the Dulong-Petit limit, *D* is the thermal diffusivity measured using a laser flash technique (Netzsch LFA467)^36^. Transport property measurements were performed within 300–600 K and the uncertainty of each was about 5%.

### Reporting summary

Further information on research design is available in the Nature Research Reporting Summary linked to this article.

## Supplementary information

Supplementary informations

Reporting Summary

## Data Availability

The all data generated or analyzed in this study are included in the published article and its Supplementary Information.
