# Supplementary material for: A record thermoelectric efficiency in tellurium-free modules for low-grade waste heat recovery
Source: Nat Commun. 2022 Jan 11;13:237. doi: 10.1038/s41467-021-27916-y (PMC8752736; doi:10.1038/s41467-021-27916-y)
Supplement: Supplementary file 1 — Supplementary informations [file 41467_2021_27916_MOESM1_ESM.pdf]

Supplementary Information

A record thermoelectric efficiency in tellurium-free modules for low-grade waste heat recovery

Zhonglin Bu[1], Xinyue Zhang[1], Yixin Hu[1], Zhiwei Chen[1], Siqi Lin[1], Wen Li[1], Chong Xiao[2] and Yanzhong Pei[1, *]

1. Interdisciplinary Materials Research Center, School of Materials Science and Engineering, Tongji Univ., 4800 Caoan Rd., Shanghai 201804, China.

2. Hefei National Laboratory for Physical Sciences at the Microscale, University of Science and Technology of China, Hefei, Anhui, 230026 P. R. China

*Email: yanzhong@tongji.edu.cn

Fabrication of thermoelectric modules:

Both n- and p-type legs were sliced to have a cross-section area of ~1.5×1.5 mm^2 with vary length. The n- and p-type legs were bridged by a copper plate at hot side using Sn-Sb-Pb solder (melting point of ~360 ℃), and the cold side were welded into a direct bonding copper plate using tin-based solder (melting point of ~210 ℃), and then alternately positioned onto an insulated alumina ceramics (Al_2O_3) with dimensions of 10×10×0.65 mm^3. This substrate is well suited for thermoelectric module due to its good thermal conductivity and electrical insulation. Detailed dimensions of different modules are shown in the Supplementary Table 1. Two Cu wires were soldered to the cold-side Cu plates to supply current and measure the terminal voltage.

Supplementary Table 1. The detailed dimensions of the modules in this work.

Module	Framework (mm^3)	N (pair)	Cross-sectional area, *A* (mm^2)	Material length, *L* (mm)	Sb or Fe layer (mm)	Ni layer (mm)
Module-1	10×10×5	7	1.5×1.5	2	0.1	0.4
Module-2	10×10×8	8	1.5×1.5	4	0.1	0.9
Module-3	10×10×8	8	1.5×1.5	4	0.1	0.9

Thermoelectric efficiency measurements:

The power output (P) and conversion efficiency (η) of the Mg_3SbBi/CdSb modules under different temperature differences were measured in vacuum (Supplementary Fig. 1)[1]. The cold-side temperature was maintained by water-cooling system. The heater, module and heat-flow meter (Cu bar) were assembled under a uniaxial pressure to improve the thermal contacts. Thermal grease (QM850) was used to improve the thermal contact between the heater and module and between the module and heat-flow meter. K-type thermocouples (Omega) were adhered to Al_2O_3 ceramics using silver paste for measuring the temperature difference between the hot (T_h) and cold (T_c) side temperatures of the module.

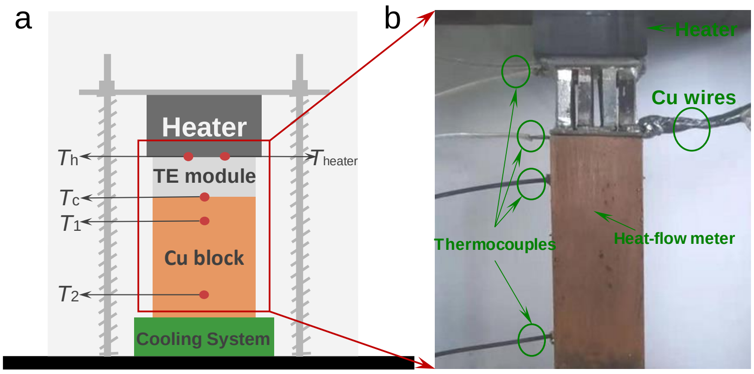

Supplementary Fig. 1. Schematic **(a)** and a typical photograph **(b)** for the setup measuring the output power and efficiency of thermoelectric module.

Two tubular heaters (Omega) were embedded in a graphite block for heating the module at hot side. Graphite was chosen for its high thermal conductivity and low thermal expansion coefficient. Circulating water cooling block along with a thermoelectric cooling plate were used to cool the temperature of the cold side. A Cu bar with a cross-sectional area identical to that of the

module (10×10 mm^2) is used as a heat-flow meter with known thermal conductivity[1] for measuring the heat flow through the thermoelectric module. Two K-type thermocouples (Omega) with a wire-diameter of only 0.6 mm for reducing heat loss were embedded and soldered to the heat-flow meter for determining the temperature difference. The distance between these two thermocouples is 17 mm.

The typical temperature differences between the hot- and cold-side of the heat-flow meter (ΔT_{Cu}) are 0.4-1.6 K (Supplementary Table 1, Supplementary Fig. 2a), depending on the temperature differences applied to the module (ΔT). The linear relationship between ΔT and ΔT_{Cu} (Supplementary Fig. 2b) nicely suggests the consistent responsivity of the heat-flow meter. In order to ensure the accuracy in determining the temperature difference of the heat-flow meter, two K-type thermocouples (Omega, wire-diameter of 0.6 mm) are buried inside the copper-bar with a further soldering, and each thermocouple takes 60 measurements for averaging (Supplementary Fig. 2a). As can be seen, even at the smallest ΔT_{Cu} of ~0.64 K, the relative standard deviation (RSD) for these 60 measurements can be ensured to be within 3%, indicating the sufficiently large signal to noise ratio.

A measurement of thermoelectric efficiency was enabled by measuring the output power (P=IV) and the heat flow in vacuum, where I is the current and V is the output voltage. By varying the load resistance and measuring the corresponding load voltage and current, the maximum output power can be obtained.

The heat flow can be obtained by:

$$Q = \frac{\kappa_{Cu} A_{Cu}}{L_{Cu}} \Delta T_{Cu} \quad , \qquad \text{S1}$$

where Q, A_{Cu}, L_{Cu}, $\Delta T_{Cu}=T_1\text{-}T_2$ and κ_{Cu} are the heat flow, cross-sectional area of the copper bar, distance between the thermocouples, temperature difference and thermal conductivity of the heat-flow meter (Cu block). Because of the small temperature difference (ΔT_{Cu}), the thermal conductivity (κ_{Cu}) is approximated as temperature independent. Therefore, the efficiency (η) can be estimated according to $\eta = P/(P+Q)$. The maximum efficiency (η_{max}) can be obtained by varying the load resistance in the circuit, and measuring the corresponding output power and heat flow. To minimize the system error, we measured each parameter (including temperature, voltage and current) for 60 times for averaging.

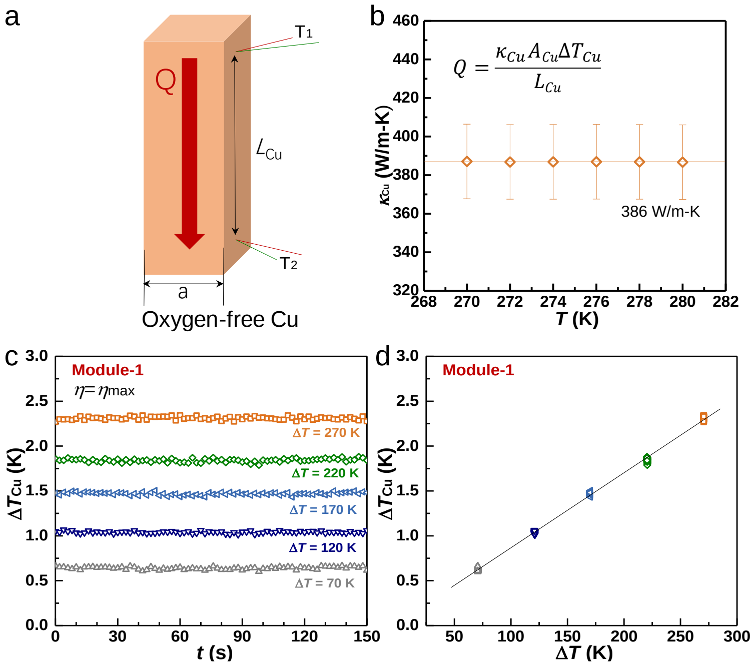

Supplementary Fig. 2. Schematic for the setup measuring heat flow **(a)**, temperature dependent thermal conductivity of oxygen-free Cu (κ_{Cu}) **(b)**, temperature differences of the heat-flow-meter ($\Delta T_{Cu}=T_1\text{-}T_2$) under different temperature differences (ΔT) applied to the module **(c)** and the relationship between **(d)**.

Supplementary Table 2. The temperature difference applied to the module (ΔT) and the corresponding temperature difference for the heat-flow meter (ΔT_{Cu}) of 60 measurements for averaging and its Relative Standard Deviation (RSD) of the heat-flow meter. The estimated uncertainty of ΔT_{Cu}, measured heat flow (Q), and conversion efficiency (η) of the module in this work.

**ΔT (K)**	**ΔT_{Cu} (K)**	**RSD (%)**	**Uncertainty of ΔT_{Cu} (%)**	**Uncertainty of Q (%)**	**Uncertainty of η (%)**
70	0.64	2.9	7.8	14.1	12.3
120	1.04	2.3	4.8	11.3	9.8
170	1.47	1.5	3.4	10.1	8.4
220	1.84	0.9	2.7	9.4	7.7
270	2.31	0.6	2.2	8.9	7.2

Estimation of heat radiation and leakage:

In this work, a control experiment is carried out to figure out the main origin leading to the discrepancy between measured and predicted heat flows. In order to measure the vertical thermal radiation between the uncovered inner surfaces of the alumina-ceramic substrates of the module, two alumina-ceramic substrates of the same size are respectively fixed to the heater (hot side of the module) and the heat-flow meter (cold side of the module) with an exact separation of 3 mm (the total length of the leg and contacts of Module-1). The cold-side temperature is kept at 280 K, and varying the hot-side temperature enables a measurement of the corresponding vertical radiative heat flows under different temperature differences. As shown in Supplementary Fig. 3**,** an exact measurement setup of Module-1 but without thermoelectric materials is used to estimate the induced vertical heat flow due to heat radiation (vertical) between the substrates of the module. This corresponds to the extreme case of a zero-filling-fraction module, and the heat flow [$Q_{\rm rad}(f=0)$] of which can be used to estimate the case of a real case of module with a filling fraction of f=0.315 for Module-1 by [$Q_{\rm rad}(f\neq 0)=Q_{\rm rad}(f=0)\times(1-f)$]. This helps understand that the discrepancy between measured and predicted heat flow increases with increasing ΔT, due to the increase in vertical heat radiation from the unfilled portion of hot-side substrate to the cold-side one at high temperatures (Supplementary Fig. 3).

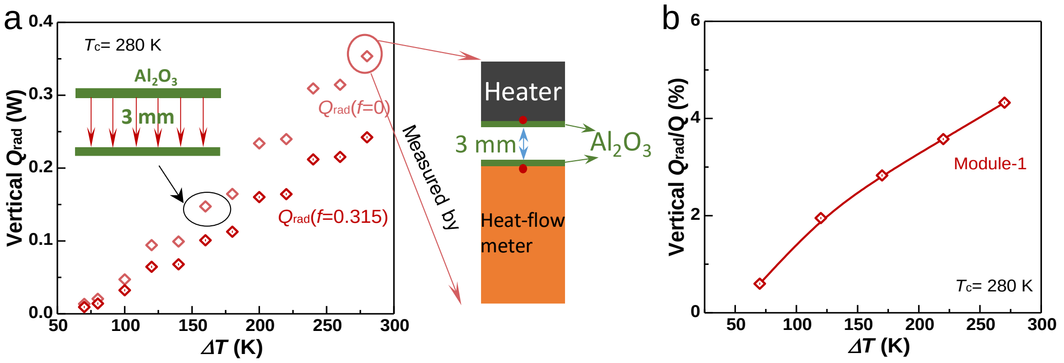

Supplementary Fig. 3. Temperature gradient dependent measured vertical radiation heat **(a, b)**.

The heat radiated from the surfaces of legs to the surroundings (horizontal) can be estimated by the following equation[2,3]:

$$Q_{rad} = \frac{\varepsilon\theta C}{\Delta T}\int_{T_c}^{T_h}(T^4 - T_S^4)\,dT \tag{S2}$$

where the $Q_{\rm rad}$ is the horizontal heat radiation, ε is the emissivity, θ is the Stefan-Boltzmann constant, C is the total outer side surface area of thermoelectric legs, ΔT is the temperature gradient, $T_{\rm h}$ is the hot-side temperature, $T_{\rm c}$ is the cold-side temperature, T is the temperature distribution, and $T_{\rm S}$ is the surrounding temperature. The emissivity of 0.5 is used according to the previous reports of thermoelectric materials[2-4]. The surrounding temperature is measured by two thermocouples in the vacuum chamber, and average (Supplementary Fig. 4). The estimated horizontal heat radiation increases with increasing temperature, leading to a heat loss of 2% at $T_{\rm h}$=550 K and $T_{\rm c}$=280 K of Module-2 with a leg length of 4 mm. Since the horizontal heat radiation is proportional to the radiative surface area, Module-1 with a shorter leg length of 2 mm reduces the heat radiation loss to be only 0.63% at the same temperature gradient of 270 K (Supplementary Fig. 4). Compared to the total heat-flow, the horizontal heat radiation loss for Module-1 is negligible. In this work, both horizontal and vertical heat radiations are taken into account for estimating the efficiency, and the corresponding maximum efficiency are listed in Supplementary Table 3. Note again that vertical radiation between unfilled module substrates leads to an overestimation in heat flow but horizontal one leads to an underestimation.

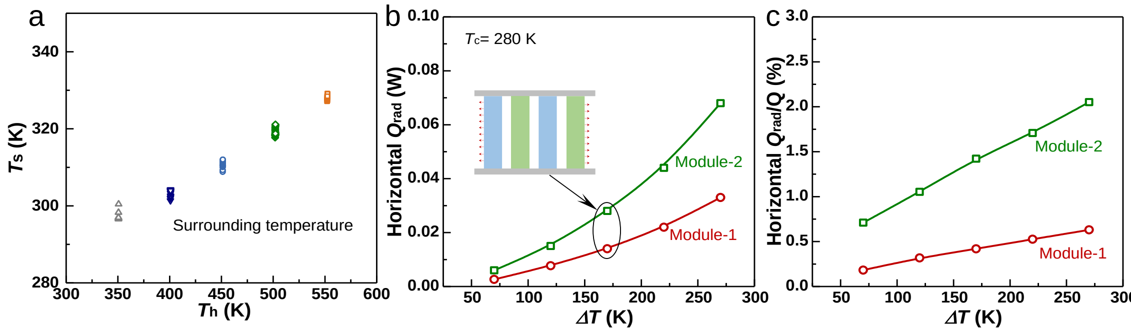

Supplementary Fig. 4. Hot-side temperature dependent surrounding temperature $T_{\rm S}$ **(a)**, temperature gradient dependent estimated horizontal radiation heat $Q_{\rm rad}$ **(b),** and its fraction to the total heat flow **(c)**.

Supplementary Table 3. Temperature gradient dependent maximum efficiency of Module-1 with various corrections considering horizontal and/or vertical heat radiations.

ΔT (K)	η_{max} (%) without considering the heat radiations	Horizontal Q_{rad} (mW)	Corrected η_{max} (%) with horizontal Q_{rad}	Vertical Q_{rad} (mW)	Corrected η_{max} (%) with vertical Q_{rad}	Corrected η_{max} (%) With both horizontal and vertical heat radiations
70	1.59	3	1.58	9	1.60	1.59
120	3.22	8	3.21	50	3.29	3.28
170	4.64	14	4.62	100	4.78	4.76
220	6.46	22	6.42	160	6.69	6.66
270	8.21	33	8.17	242	8.57	8.52

Heat leakages can be important sources causing measurement uncertainties. Indeed, we used thermocouples and Cu wires with as thin as possible diameters. Thermocouples/wires shown in Supplementary Fig. 1b come with insulation/shielding accessories, which make them look bigger than they actually are. In this work, note that the heat-flow meter locates underneath the cold-side of the module, the temperature of which is actually lower than the surrounding temperature (Supplementary Fig. 3). Nevertheless, heat transferred between the heat-flow meter and the surroundings is estimated to be only about 0.9% of the total heat Q. Even operating at a hot-side temperature of 550 K, additional heat transferred due to the thermal conduction of thermocouples, wires attached to the module and heat-flow meter, is estimated to be not greater than 2% of Q according to the Fourier's law. Due to the small contribution, this work does not take into account the effect of heat transferred by these wires and thermocouples shown in Supplementary Fig. 1b.

Uncertainty analyses:

In this work, an oxygen-free Cu-bar is used as the heat-flow meter. The thermal conductivity (κ) of Cu is determined by $\kappa = dCpD$, where d, Cp, D are density, heat capacity and thermal diffusivity. The density was estimated by mass/volume and the thermal diffusivity was measured by a laser flash technique (Netzsch LFA457), the uncertainty in the measurement of κ is about 5% (Supplementary Fig. 2). The uncertainties of cross-area (A_{Cu}) and length (L_{Cu}) are less than 1%. At given steady temperature gradients applied to the module, the corresponding $\Delta T_{Cu}=T_1-T_2$ fluctuate within 2~8% (Supplementary Table 2). Note that the uncertainty of ΔT_{Cu} decreases with increasing temperature gradient of the module (ΔT), leading to a small uncertainty of ~2% at the high-efficiency temperature gradient of ΔT=270 K. Moreover, to verify the accuracy and sensitivity of the heat-flow meter, intentionally varied hot-side temperature T_h of the module lead to corresponding synchronous variations in T_1 and T_2 of the heat meter, however, $\Delta T_{Cu}=T_1-T_2$ easily gets stabilized (Supplementary Fig. 5). No matter how the temperature gradient of the module is applied, steady or fluctuated, ΔT_{Cu} shows a rough linear increase with increasing ΔT (Supplementary Fig. 5f and Supplementary Fig. 2d). The open-circuit voltage (V_{oc}) responds quickly to the hot-side temperature (Supplementary Fig. 6), further suggests the high responsivity of the measurement setup. In addition, the nice agreement in module efficiencies measured either by a steady-temperature-gradient or by oscillating-temperature-gradient techniques, confirms the excellent responsivity of the measurement setup (Supplementary Fig. 26 and Supplementary Fig. 27). All these features confirm the overall good stability/reliability/sensitivity of the heat-flow meter (Supplementary Fig. 2c).

Therefore, the overall uncertainty of the efficiency measurement in this work is about 7~12%, depending on the temperature gradients applied to the module, and a larger temperature gradient tends to reduces the uncertainty (Supplementary Table 2).

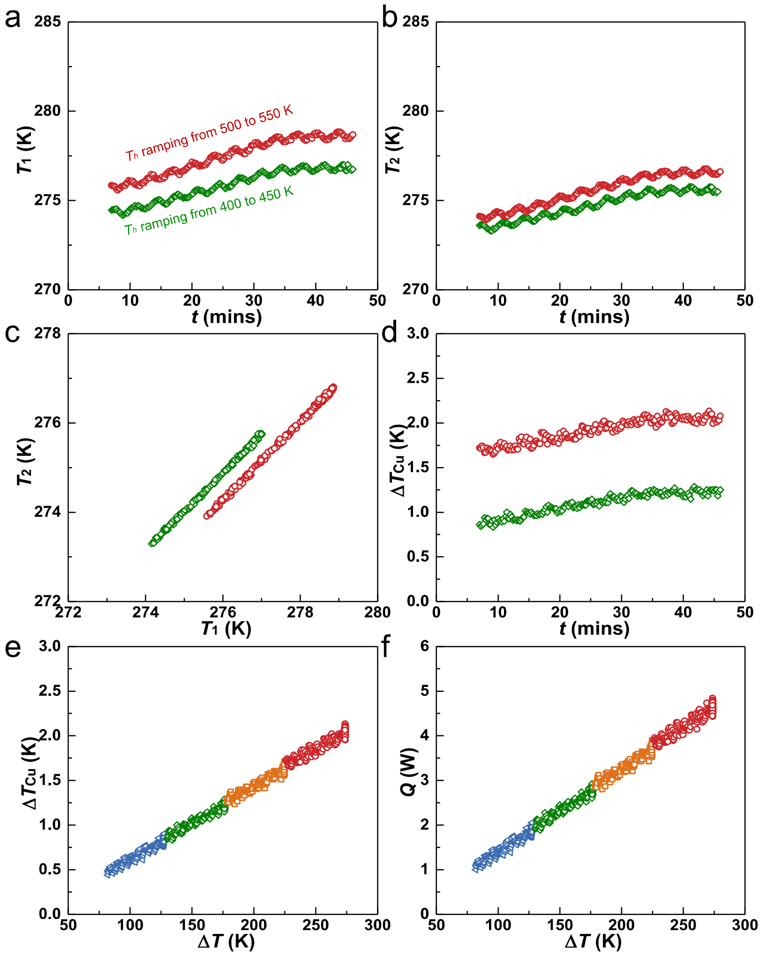

Supplementary Fig. 5. Temperature responsivity of the heat-flow meter under intentionally fluctuated heating process.

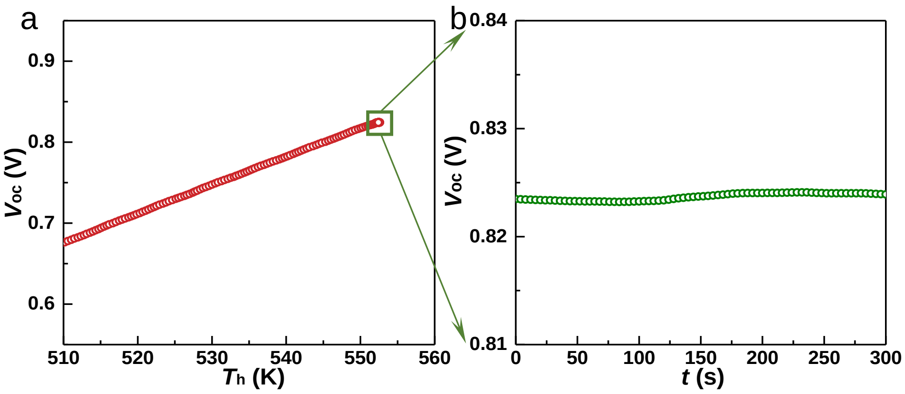

Supplementary Fig. 6. Hot-side temperature (T_h) **(a)** and time (t) **(b)** dependent open-circuit voltage (V_{oc}).

Prediction of heat-flow:

In this work, the heat-flow of a thermoelectric module is estimated by a simplified analytical one-dimensional model[5]. The internal resistance of the module (R_{in}) is:

$$R_{in} = R_{mateials} + R_{contacts} = N\left(\frac{L}{A_p}\overline{\rho_p} + \frac{L}{A_n}\overline{\rho_n}\right) + R_{contacts} \quad \text{S3}$$

where $R_{materials}$ is the total resistance of the thermoelectric materials, $R_{contact}$ is the total contact resistance, N is the number of pairs of couples, L is the length of each thermoelectric leg, A_p and A_n are respectively the cross-sectional areas of p-leg and n-leg, $\overline{\rho}_p$ and $\overline{\rho}_n$ are respectively the average resistivity of p-type material and n-type material.

The open-circuit voltage (V_{oc}) and the output power (P) of the module are:

$$V_{oc} = N(T_h - T_c)(\overline{S_p} - \overline{S_n}) \quad \text{S4}$$

$$P = V_{oc}I - R_{in}I^2 \quad \text{S5}$$

where T_h and T_c are respectively the hot-side and cold-side temperatures, $\overline{S}_p$ and $\overline{S}_n$ are respectively the average Seebeck coefficient of p-type and n-type materials, I is the current.

The open-circuit heat-flow (Q_{oc}) of the module is:

$$Q_{oc} = N(\frac{A_p}{L}\overline{\kappa_p} + \frac{A_n}{L}\overline{\kappa_n}) \quad \text{S6}$$

where $\overline{\kappa}_p$ and $\overline{\kappa}_n$ are respectively the average thermal conductivity of p-type and n-type materials.

The input heat at the hot side (Q_{input}) of the module:

$$Q_{input} = Q_{oc} + N[S_p(T_h) - S_n(T_h)]T_hI - R_{in}I^2/2 - N(\beta_p + \beta_n)T_hI/2 \quad \text{S7}$$

where β_p and β_n are combined coefficient of p-type and n-type materials, respectively.

$$\beta_n = \frac{T_c}{T_h}[S_n(T_c) - \overline{S_n}] + [\overline{S_n} - S_n(T_h)] \quad \text{S8}$$

$$\beta_p = \frac{T_c}{T_h}[\overline{S_p} - S_p(T_c)] + [S_p(T_h) - \overline{S_p}] \quad \text{S9}$$

The heat-flow at the cold-side of the module (Q) can be estimated by:

$$Q = Q_{input} - P \quad \text{S10}$$

The prediction of Q is shown in Supplementary Fig. 24b.

Calibration of the module measurement system:

In this work, the measurement accuracy has also been double checked with the results measured by the commercial instruments from both material and module levels.

For the module level, a commercial Bi_2Te_3 module HT-01702 from TECooler Technology[6] is used as the standard module for the cross-check of the measurement accuracy. The dimensions of the commercial Bi_2Te_3 module are 9.8×9.8×4.4 mm^3. The module's properties include voltage, current and heat-flow are measured at different temperature gradients by the different apparatus (Supplementary Fig. 7 and Supplementary Fig. 8). The output power $P=VI$ measured by our setup is distinguishable to that by Mini-PEM (Advance Riko, Japan), indicating the nice accuracy here in electrical properties measurements. More importantly, the heat-flow (Q) measured by this system is also highly comparable to that by Mini-PEM. This leads the maximum conversion efficiencies (η_{max}) of the commercial Bi_2Te_3 module measured by different apparatus and that disclosed by TECooler Technology to be almost the same, ensuring the accuracy of the system here for efficiency measurements (Supplementary Fig. 9).

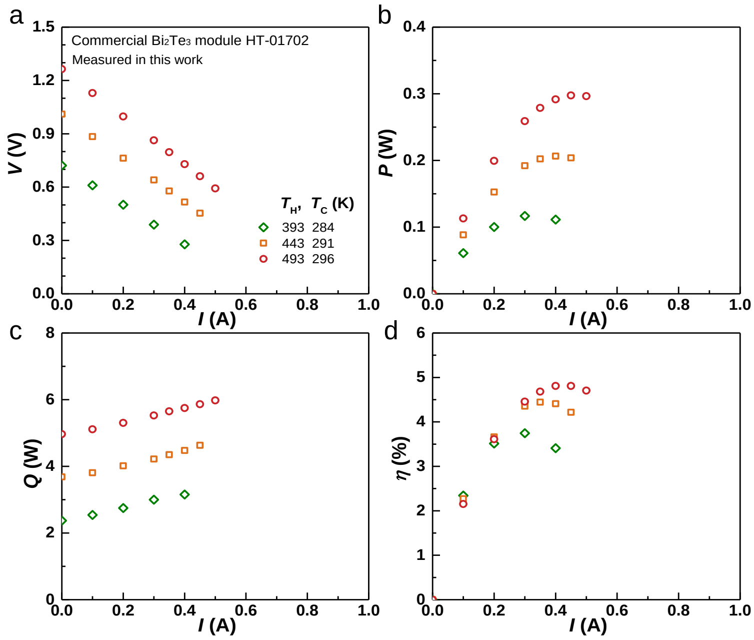

Supplementary Fig. 7. Current I dependent output voltage V **(a)**, power P **(b)**, heat flow Q **(c)**, and conversion efficiency η **(d)** under different temperature differences for commercial Bi_2Te_3 module HT-01702 measured in this work.

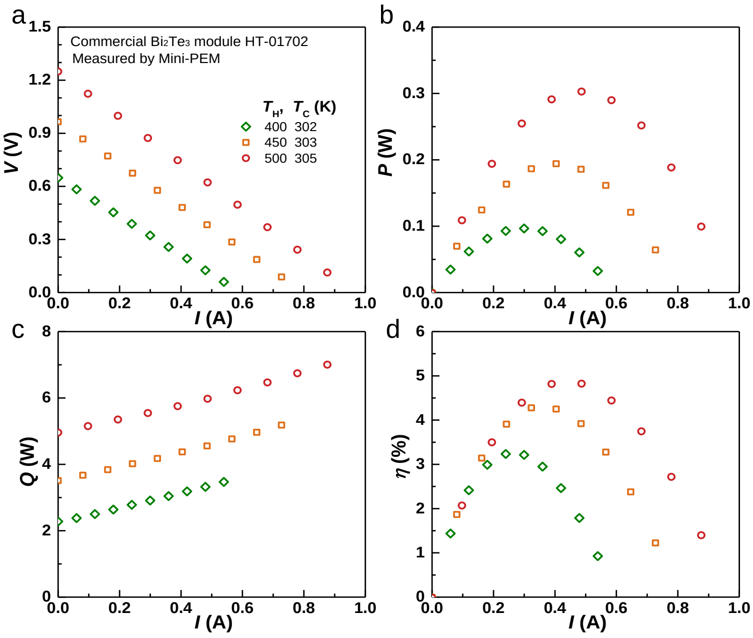

Supplementary Fig. 8. Current I dependent output voltage V **(a)**, power P **(b)**, heat flow Q **(c)**, and conversion efficiency η **(d)** under different temperature differences for commercial Bi_2Te_3 module HT-01702 measured by Mini-PEM.

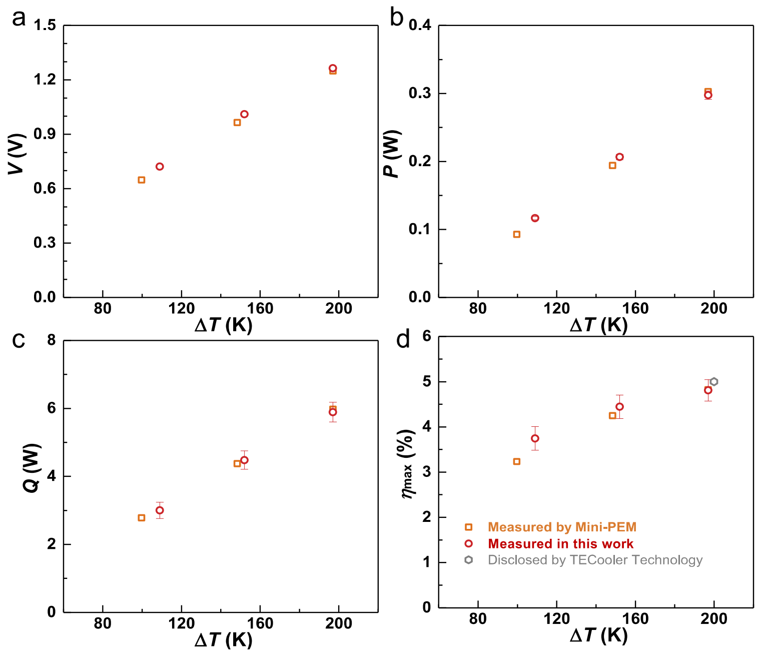

Supplementary Fig. 9. Temperature gradient dependent open-circuit voltage V **(a)**, power P **(b)**, heat flow Q **(c)**, and maximum efficiency η_{max} **(d)** for commercial Bi_2Te_3 module HT-01702 measured in this work and by Mini-PEM, with a comparison to that disclosed by TECooler Technology. The error bars represent the measurement uncertainties in this work.

For the materials level, the average thermal conductivity (κ_{avg}) of p-type CdSb and n-type Mg_3SbBi alloys is measured by a laser flash technique (Netzsch LFA467) and the steady-state technique using the exact heat-flow meter. The dimensions of the p- and n-type materials are $10\times10\times2.1$ mm^3 and $10\times10\times2.3$ mm^3, respectively. As shown in Supplementary Fig. 10, the laser flash method tends to show a lower thermal conductivity than that measured by a steady-state technique, which was indeed confirmed by our measurements in both n- and p-type materials (red data points by LFA467 locating slightly underneath those by heat-flow meter). Furthermore, we also corrected the measurement results by the steady-state technique (using the heat-flow meter), taking into account the effect of horizontal thermal radiations (absence of vertical ones in this measurement). Note that the steady-state technique (using the heat-flow meter) leads to a higher thermal conductivity than that measured by the laser flash method in both n- and p-type materials (Supplementary Fig. 10).

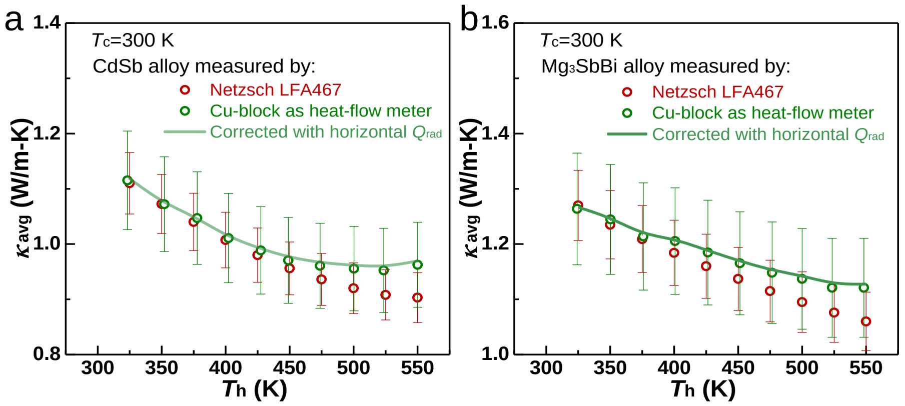

Supplementary Fig. 10. Hot-side temperature dependent average thermal conductivity (κ_{avg}) measured by Netzsch LFA467 and the heat-flow meter of p-type CdSb alloy **(a)** and n-type Mg_3SbBi alloy **(b)**. The error bars represent the measurement uncertainties in this work.

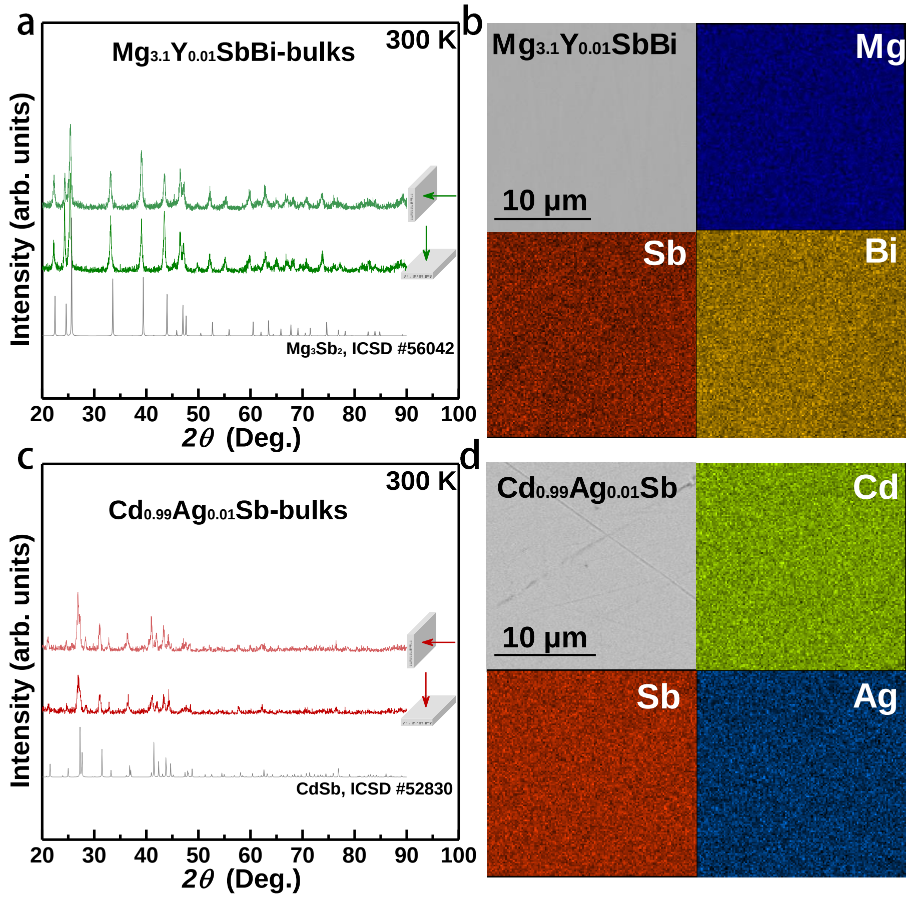

Supplementary Fig. 11. Room temperature XRD patterns **(a, c)**, back-scattering scanning electron microscope (SEM) images and corresponding energy dispersive X-ray spectroscopy (EDS) compositional mapping **(b, d)** for n-type $Mg_{3.1}Y_{0.01}SbBi$ and p-type $Cd_{0.99}Ag_{0.01}Cd$.

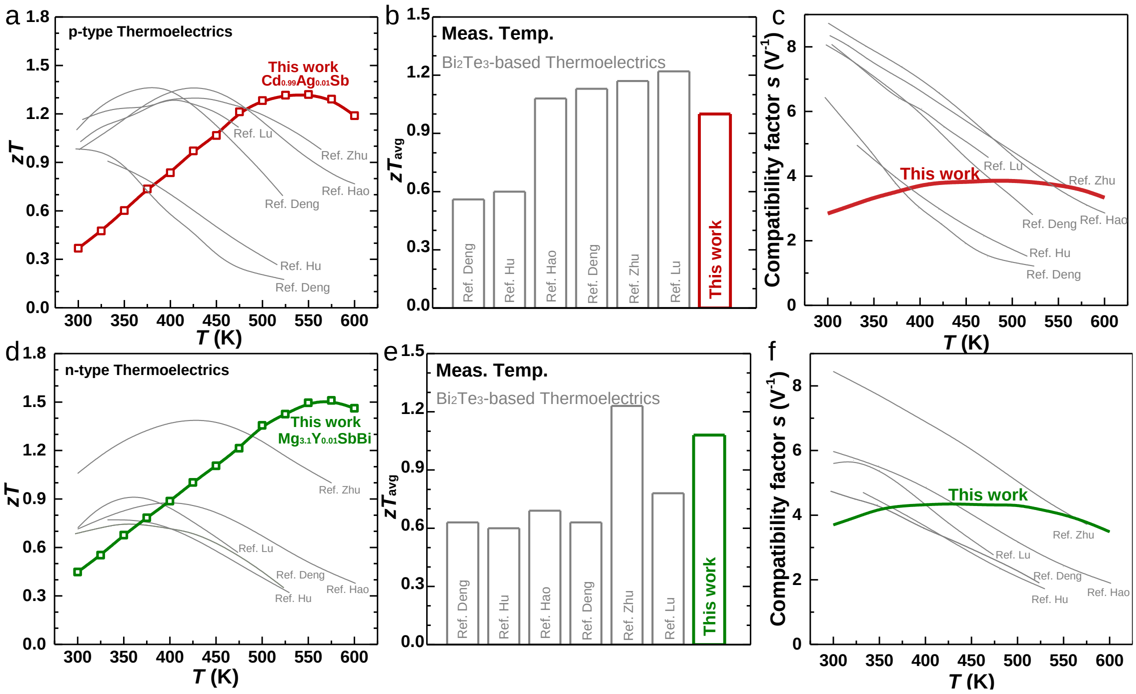

Supplementary Fig. 12. Temperature dependent thermoelectric figure of merit zT **(a, d)** and its corresponding average zT_{avg} **(b, e)** and compatibility factor **(c, f)** for p-type **(a, b, c)** and n-type **(d, e, f)** thermoelectrics, respectively, with a comparison to literature results of Bi_2Te_3-based thermoelectrics assembled for modules[7-11], and the corresponding efficiencies are shown in **Fig. 5** and **S23**.

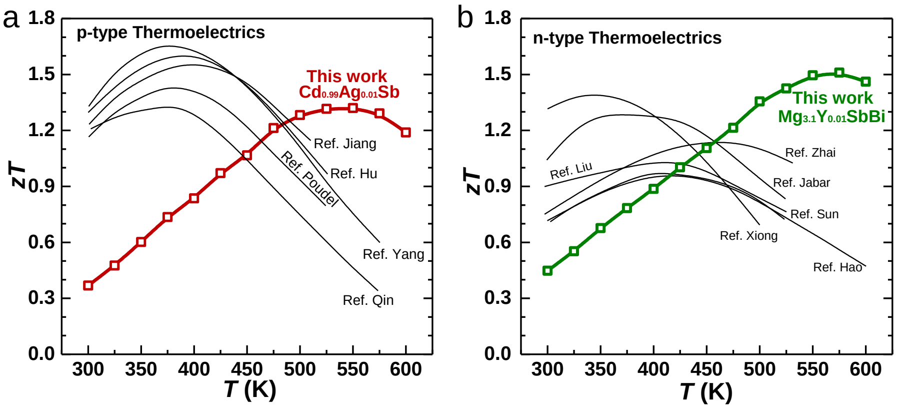

Supplementary Fig. 13. Temperature dependent figure of merit *zT* of p-type CdSb **(a)** and n-type Mg_3SbBi **(b)** with a comparison to other high-performance Bi_2Te_3-based thermoelectrics but not assembled for modules[12-22].

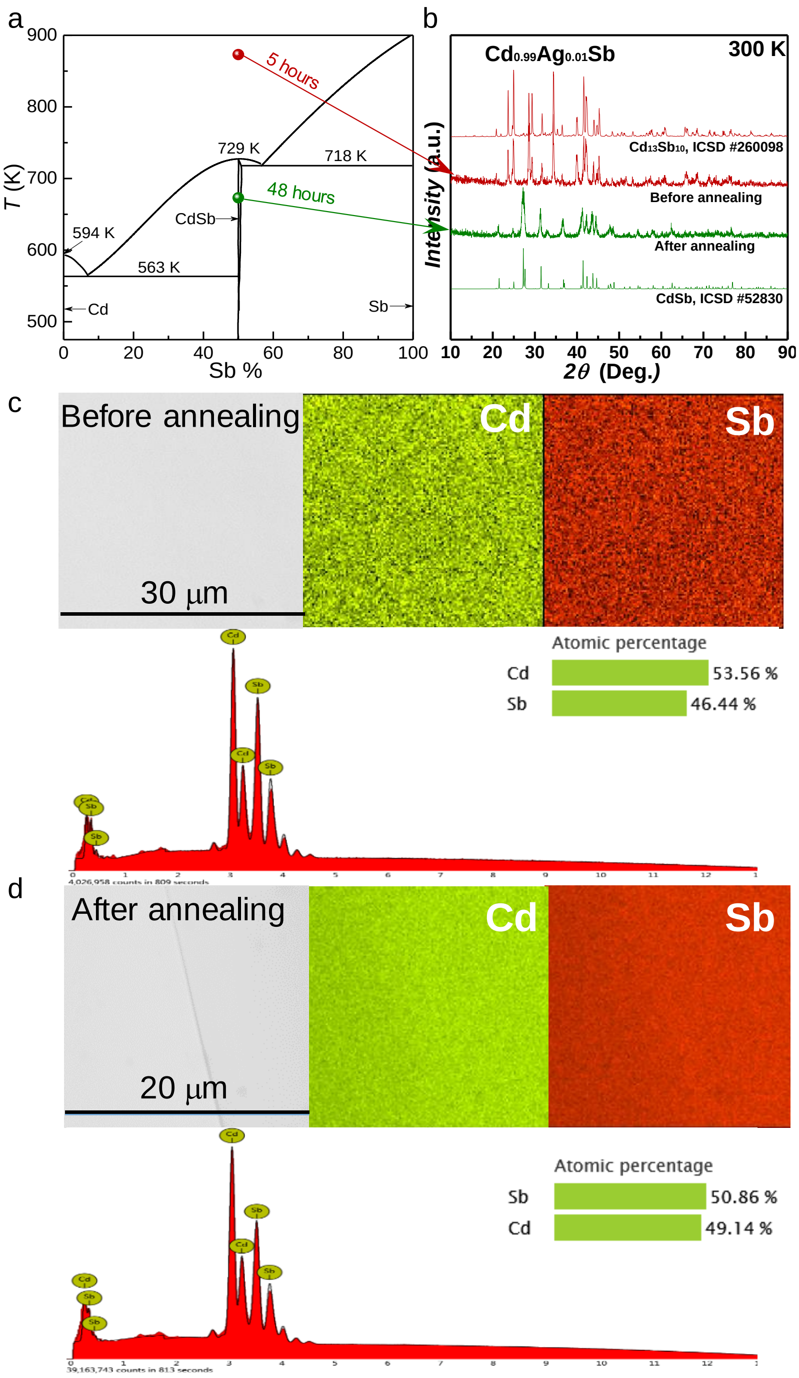

Supplementary Fig. 14. Phase diagram of binary Cd-Sb system **(a)**, powder XRD patterns for CdSb before and after a 673 K annealing for 48 hours **(b)**, and backing-scattering SEM images and EDS compositional mapping for CdSb before **(c)** and after annealing **(d)**.

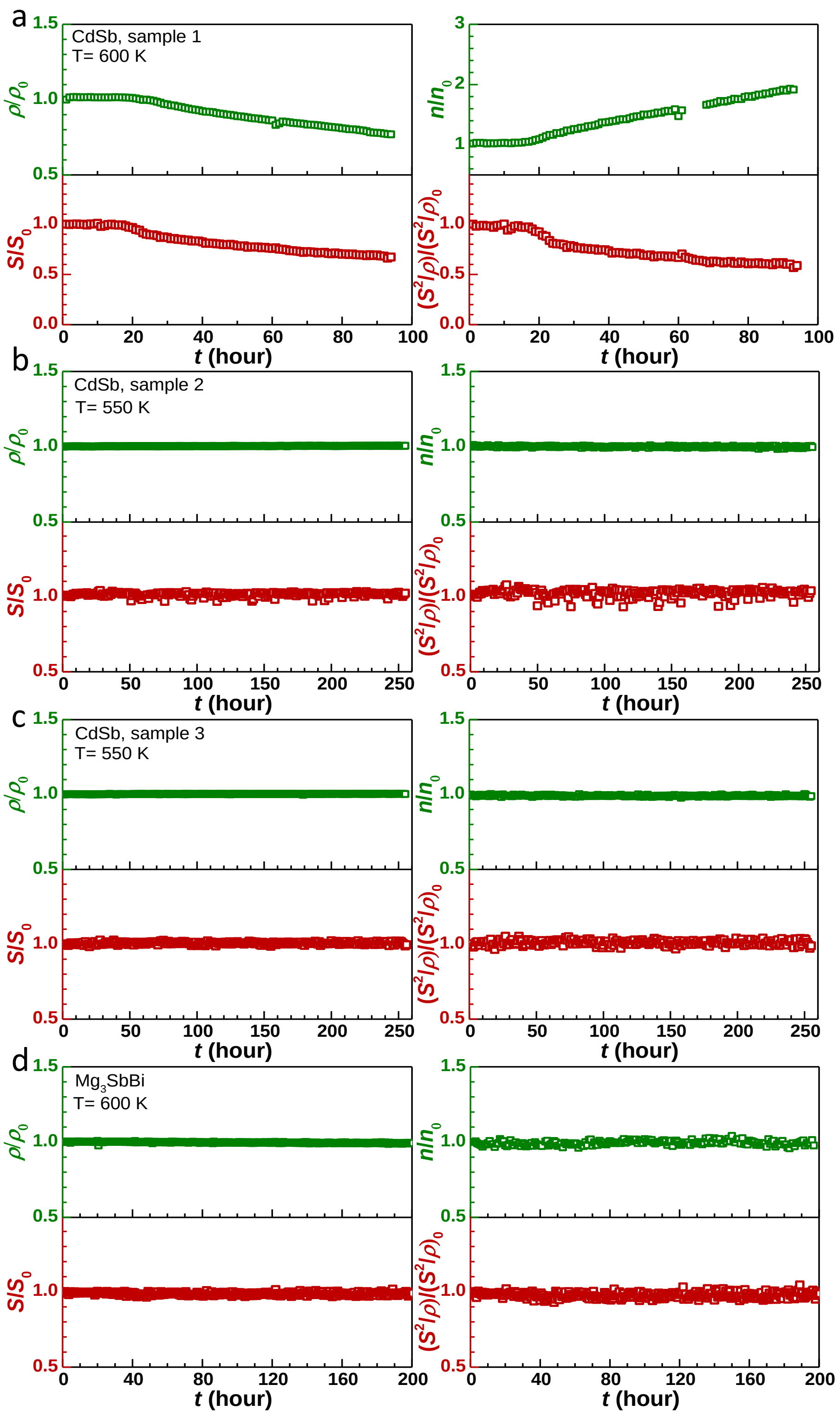

Supplementary Fig. 15. Long-term measurement of thermoelectric properties ρ/ρ_0, n/n_0, S/S_0 and $(S^2/\rho)/(S^2/\rho)_0$ for p-type CdSb **(a, b,** and **c)** and n-type Mg_3SbBi **(d)**.

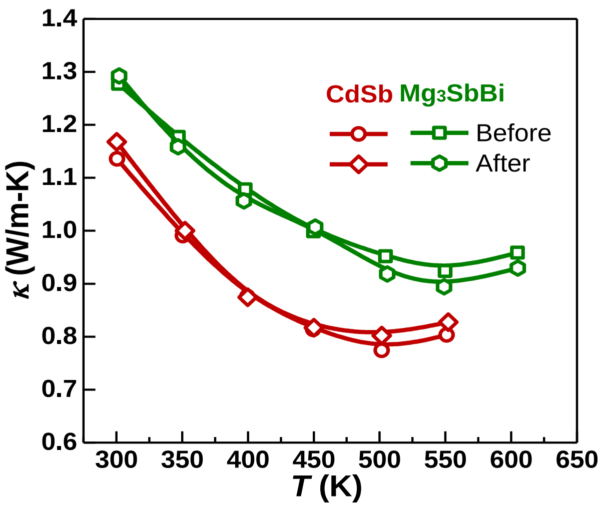

Supplementary Fig. 16. Temperature dependent thermal conductivity before and after a 200~250-hour aging of CdSb at 550 K and Mg_3SbBi at 600 K.

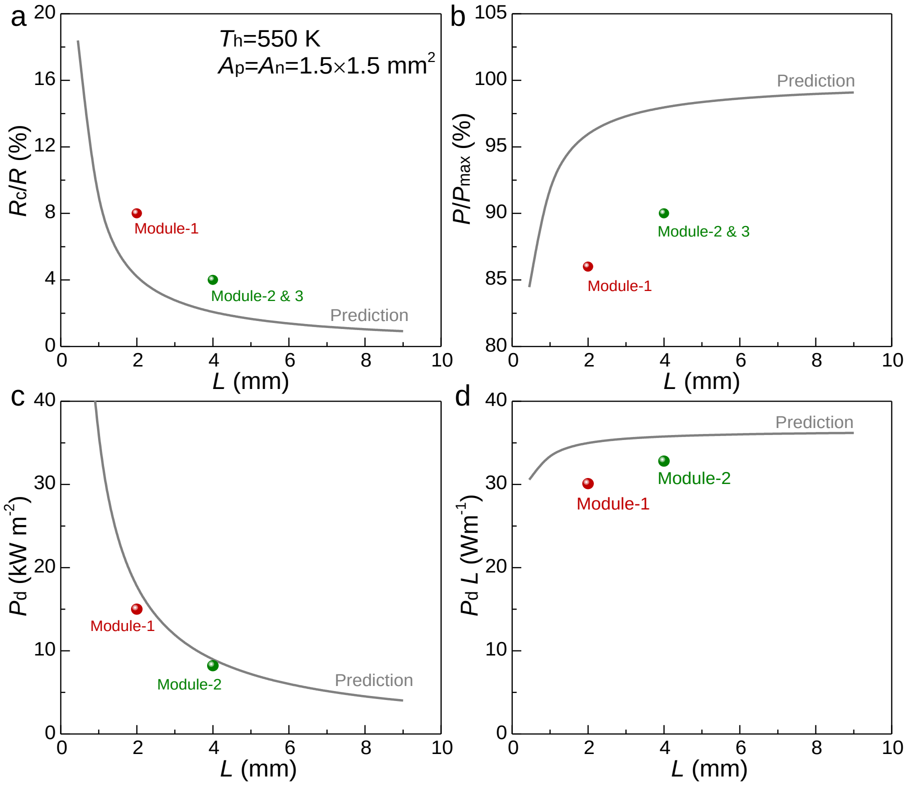

Supplementary Fig. 17. Interfacial contact resistance accounts for the total resistance (R_c/R) **(a)**, the corresponding reduction in output power (P/P_{max}) **(b)**, the output power density (P_d) **(c)**, and $P_d \times L$ **(d)** as a function of leg length of thermoelectric materials. The curves show the predictions according to the properties of thermoelectric materials and the contact resistance.

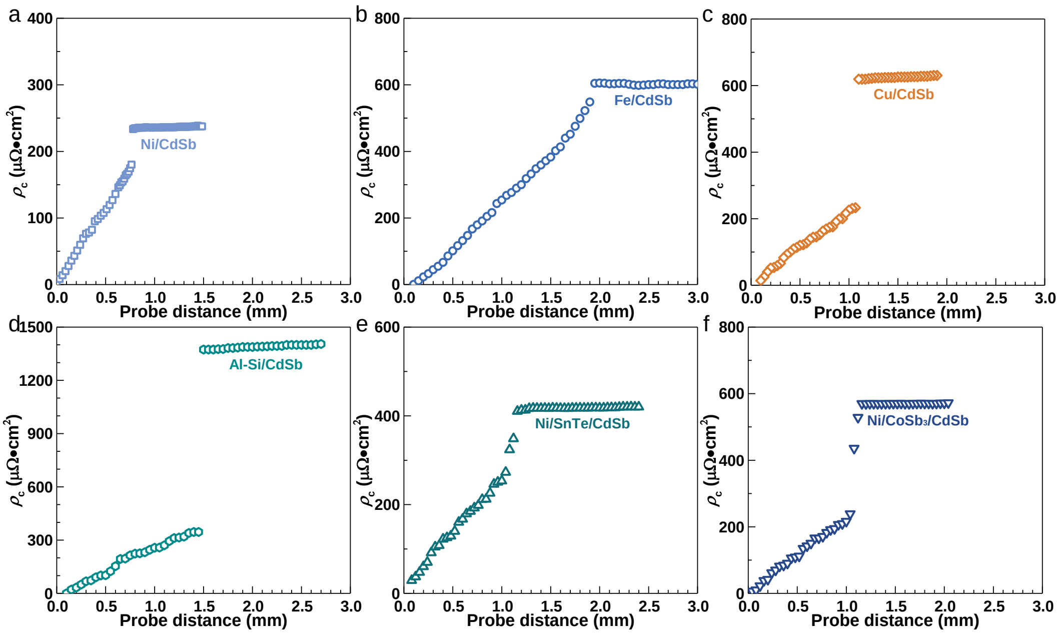

Supplementary Fig. 18. Interfacial contact resistivity for different CdSb/metal interfaces.

Supplementary Table 4. Interfacial contact resistivity for different CdSb/metal interfaces.

**Electrode/barrier/TE materials**	**ρ_c(μΩ cm^2)**
Ni/CdSb	52
Fe/CdSb	57
Cu/CdSb	387
Al-Si/CdSb	1026
Ni/SnTe/CdSb	156
Ni/$CoSb_3$/CdSb	196
Ni/Sb/CdSb	30

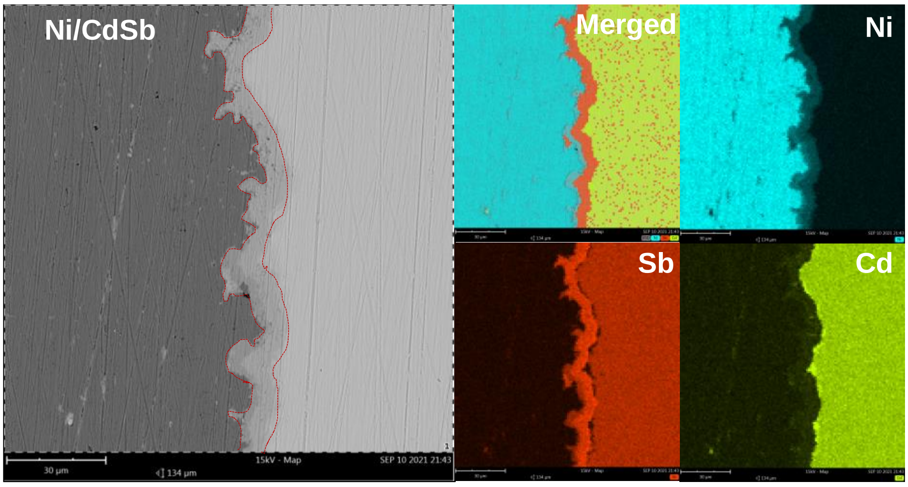

Supplementary Fig. 19. SEM images and EDS mapping of Ni/ CdSb after 14-days aging at temperature of 550 K.

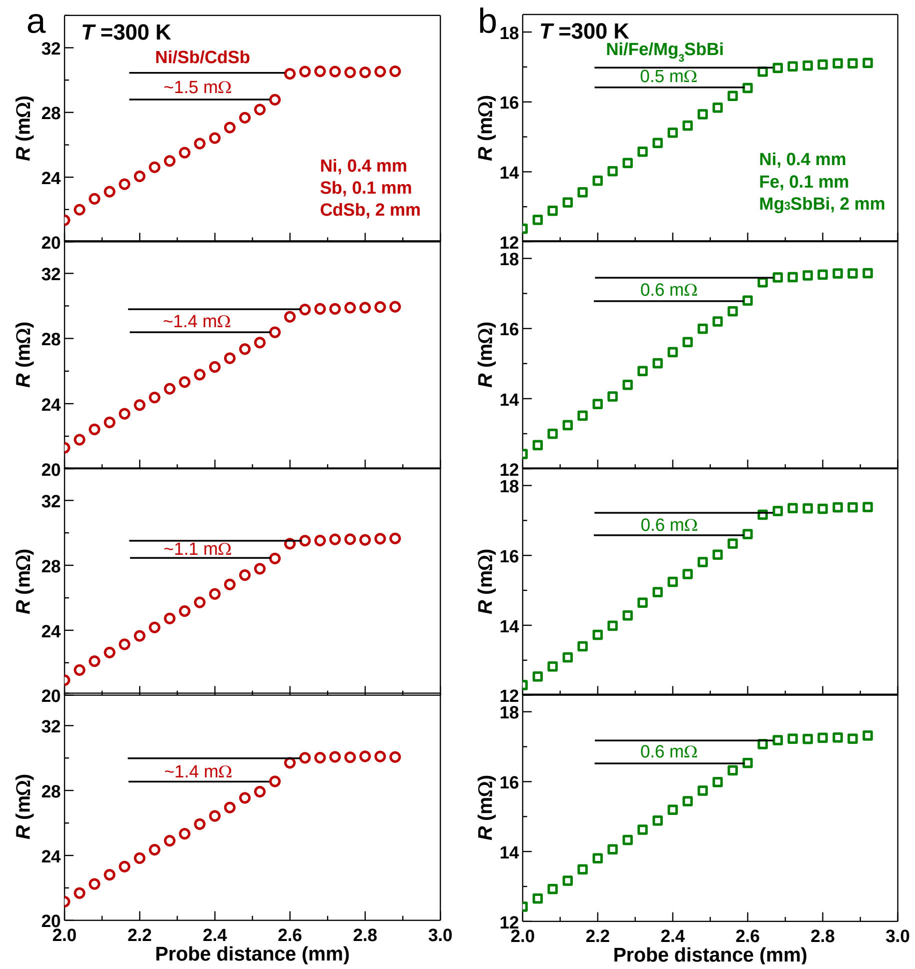

Supplementary Fig. 20. Line scanning of resistance across the Ni/Sb/CdSb **(a)** and Ni/Fe/Mg_3SbBi **(b)** interfaces of different legs.

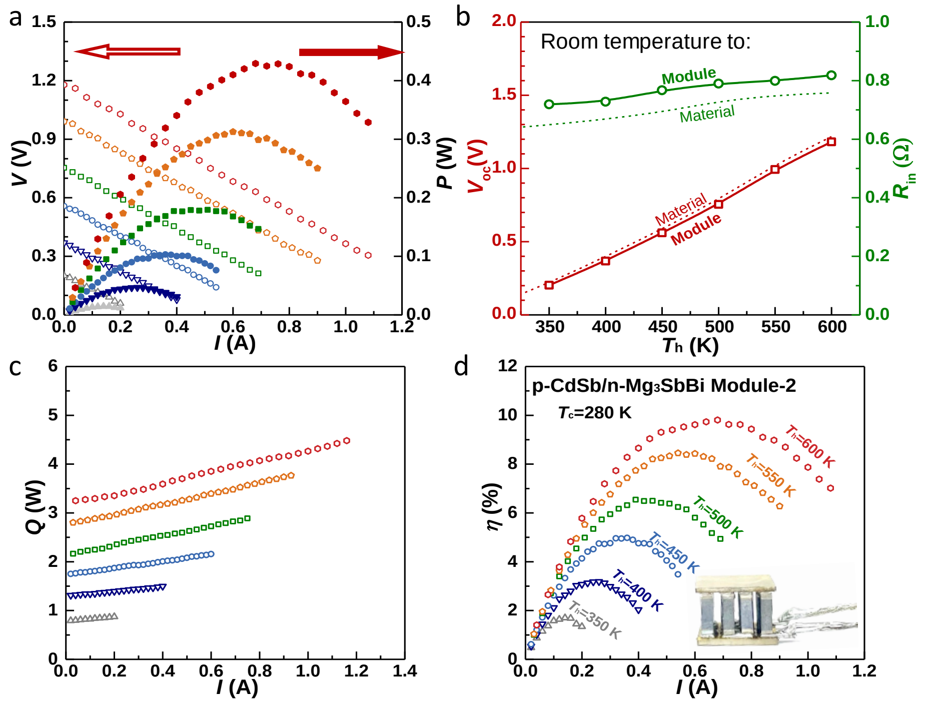

Supplementary Fig. 21. Current I dependent output voltage V and power P **(a)**, open-circuit voltage V_{oc} and internal resistance R_{in} **(b)**, heat flow **(c)**, and conversion efficiency η **(d)** under different temperature gradient for Mg_3SbBi/CdSb thermoelectric Module-2.

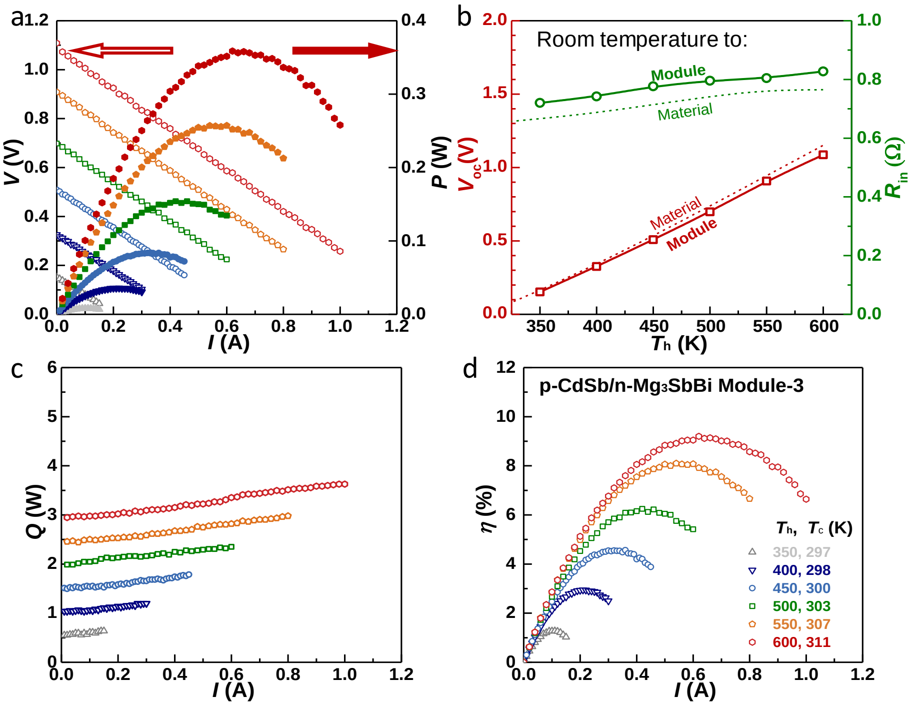

Supplementary Fig. 22. Current I dependent output voltage V and power P **(a)**, open-circuit voltage V_{oc} and internal resistance R_{in} **(b)**, heat flow **(c)**, and conversion efficiency η **(d)** under different temperature gradient for Mg_3SbBi/CdSb thermoelectric Module-3.

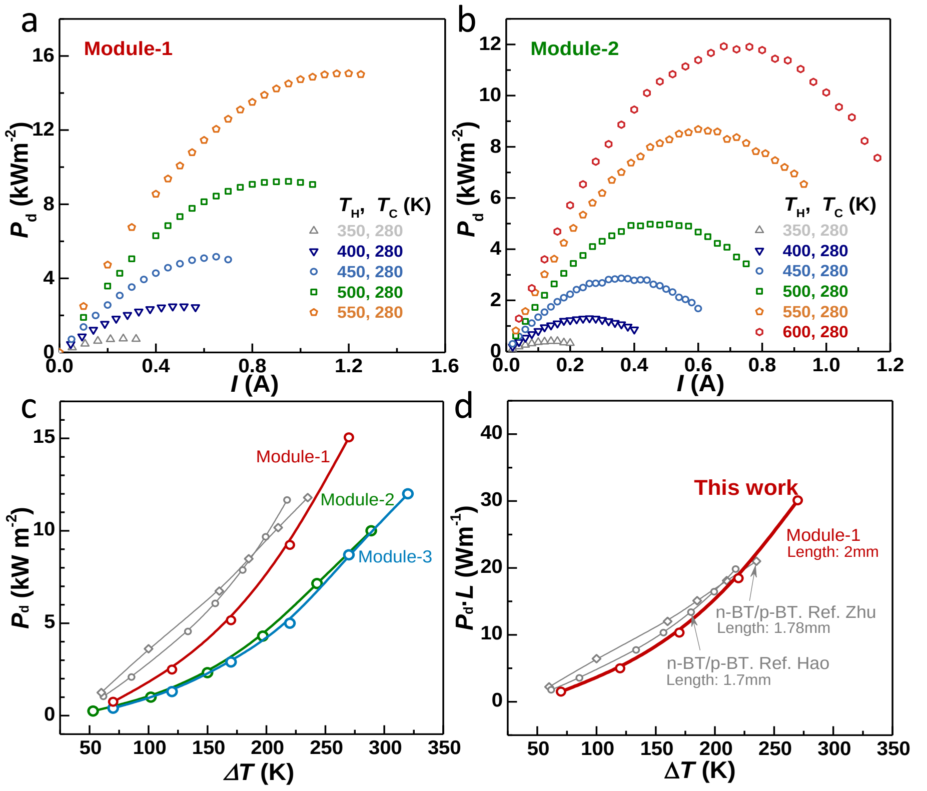

Supplementary Fig. 23. Current dependent power density (P_d) for Module-1 **(a)** and Module-2 **(b)**, temperature gradient dependent maximum power density (P_d) **(c)** and specific maximum power density ($P_d \times L$) **(d)** of the modules in this work with a comparison to literature results of Bi_2Te_3-modules[7,8].

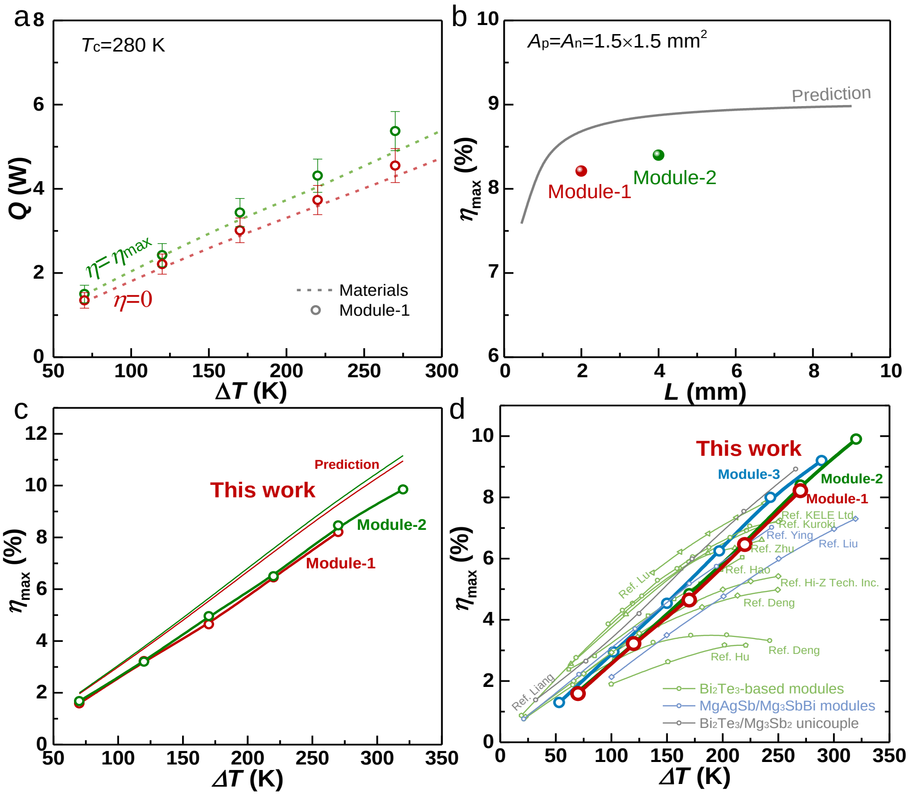

Supplementary Fig. 24. Temperature gradient dependent measurement and prediction of the heat-flow Q at η=0 and $\eta=\eta_{max}$ **(a)**, the maximum efficiency $\eta_{\max}$ as a function of the length of thermoelectric materials **(b)**, and $\eta_{\max}$ **(c and d)** for the CdSb/Mg_3SbBi modules. Literature results of Bi_2Te_3-modules and MgAgSb/Mg_3SbBi-modules are included for a comparison[2,7-11,23-27]. The error bars represent the measurement uncertainties in this work.

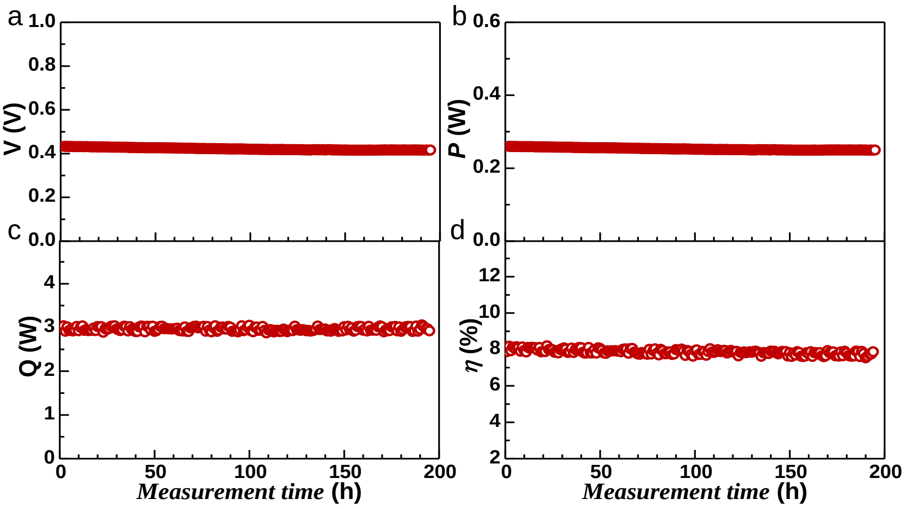

Supplementary Fig. 25. Stable output voltage (V) **(a)**, output power (P) **(b)**, heat flow (Q) **(c)** and efficiency (η) **(d)** of CdSb/Mg_3SbBi thermoelectric module at a hot-side temperature of 555 K and a cold-side temperature of 308 K.

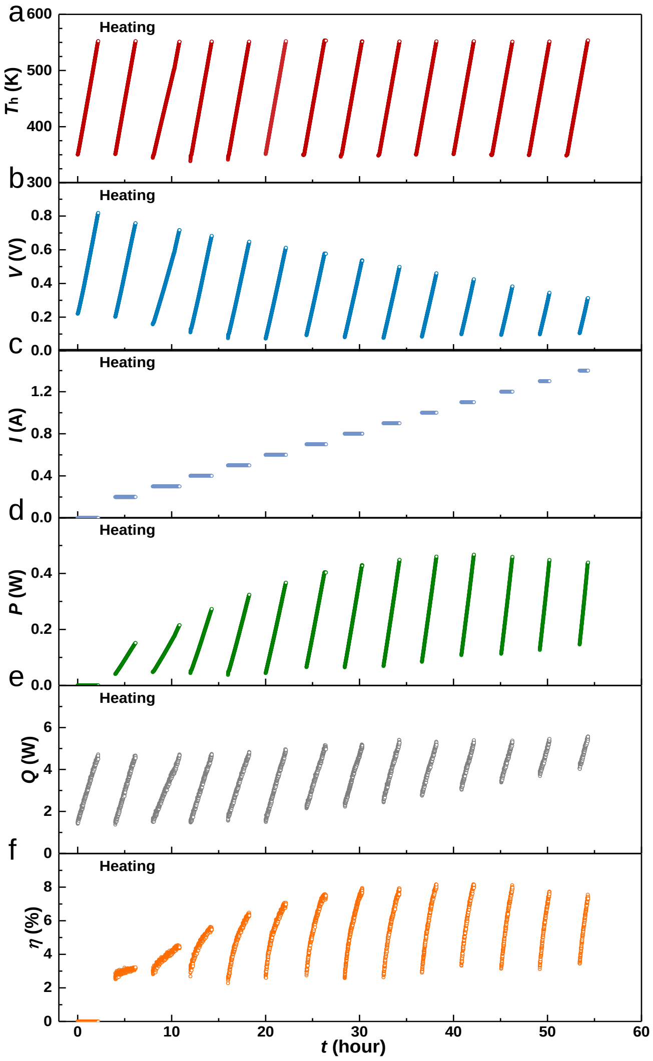

Supplementary Fig. 26. Time dependent hot-side temperature T_h **(a)**, output voltage V **(b)**, current I **(c)**, output power P **(d)**, heat flow Q **(e)** and efficiency η **(f)** of Module-1 with different load resistance.

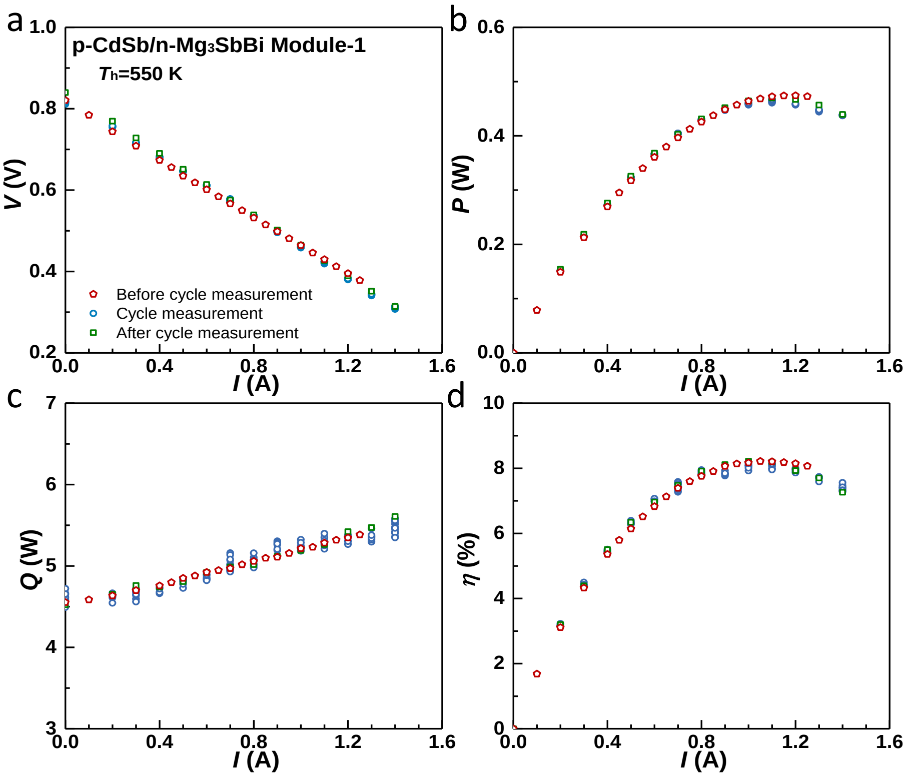

Supplementary Fig. 27. Current I dependent output voltage V **(a)**, output power P **(b)**, heat flow Q **(c)** and efficiency η **(d)** of Module-1 before and after cycle measurement.

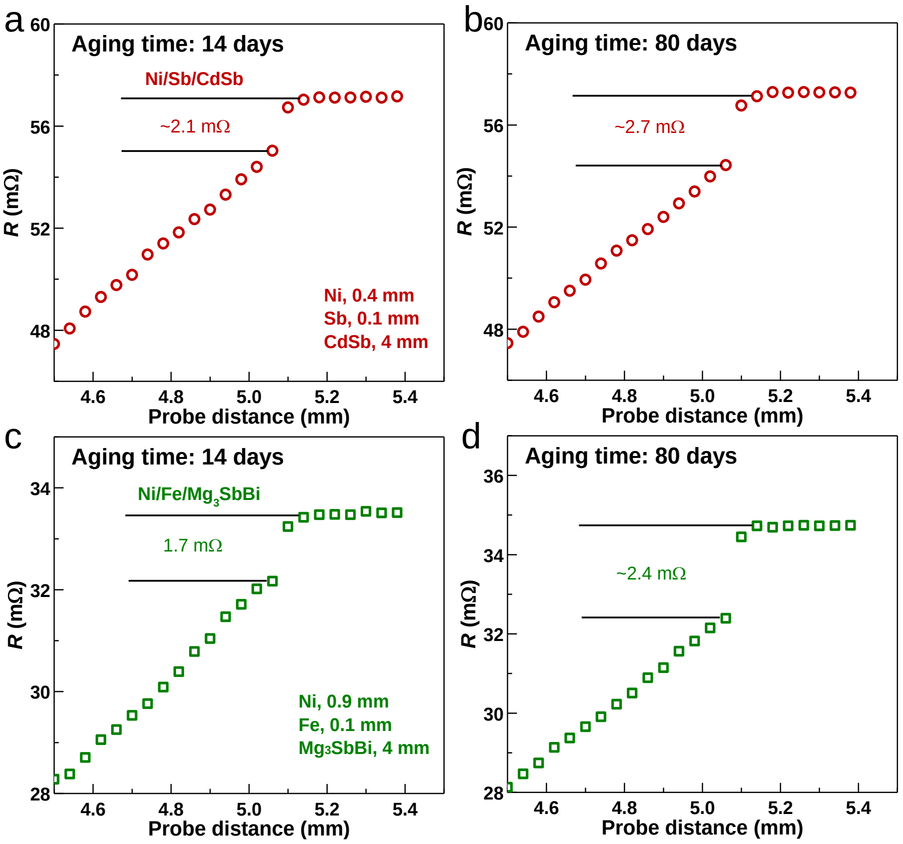

Supplementary Fig. 28. Line scanning of resistance across the Ni/Sb/CdSb **(a)** and Ni/Fe/Mg_3SbBi **(b)** interfaces after long-term aging at 550 K.

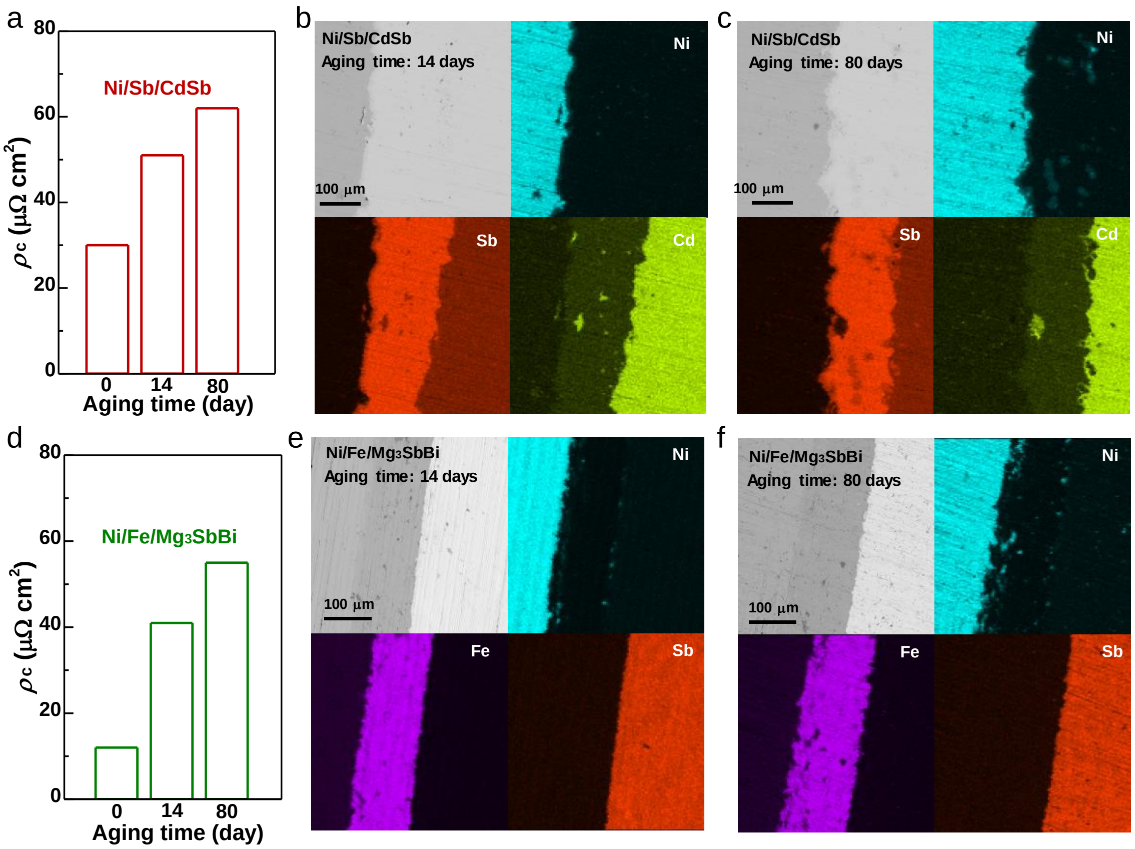

Supplementary Fig. 29. Interfacial contact resistivity after different aging time at 550 K and the corresponding SEM images and EDS mapping of Ni/Sb/CdSb **(a, b, c)** and Ni/Fe/Mg_3SbBi **(d, e, f)** interfaces.

Supplementary Table 5. The abundance (ppm%)[28], yield (t/year)[28] and price ($/kg)[29,30] of elements, the material density and the maximum efficiency (η_{max}) of different modules[7] using a heat source of ~550 K.

Element	Abundance (ppm%)	Yield(2016) (t/year)	Price ($/kg)	Material	Density (g/cm^3)	Price ($/kg)	Module	η_{max} (T_h~550 K)
Cd	0.15	23000	15	CdSb	6.8	4.37	p-CdSb/n-Mg_3SbBi	8%
Mg	23300	27700000	15.15	Mg_3SbBi	4.9	5.24		
Sb	0.2	130000	40.75					
Bi	0.0085	10200	37.5	$Bi_{0.5}Sb_{1.5}Te_3$	6.9	47.4	p-BT/n-BT	6.6%
Te	0.001	2200	460	$Bi_2Te_{2.7}Se_{0.3}$	7.8	36.28		
Se	0.05	2200	117.5					

Supplementary references

1 Bu, Z. *et al.* Realizing a 14% single-leg thermoelectric efficiency in GeTe alloys. *Science Advances* **7**, eabf2738, (2021).

2 Liang, Z. *et al.* High thermoelectric energy conversion efficiency of a unicouple of n-type Mg3Bi2 and p-type Bi2Te3. *Materials Today Physics* **19**, 100413 (2021).

3 Kraemer, D. *et al.* High thermoelectric conversion efficiency of MgAgSb-based material with hot-pressed contacts. *Energ Environ Sci* **8**, 1299-1308, (2015).

4 Zhu, Q. & Ren, Z. A double four-point probe method for reliable measurement of energy conversion efficiency of thermoelectric materials. *Energy* **191**, 116599 (2020).

5 Alleno, E. *et al.* A thermoelectric generator based on an n-type clathrate and a p-type skutterudite unicouple. *physica status solidi (a)* **211**, 1293-1300 (2014).

6 Technology, T. *TEC-HT Series*, <http://www.tecooler.com/en/products/tec.teg.html > (accessed April, 2021).

7 Zhu, B. *et al.* Realizing record high performance in n-type Bi2Te3-based thermoelectric materials. *Energ Environ Sci* **13**, 2106-2114 (2020).

8 Hao, F. *et al.* High efficiency Bi2Te3-based materials and devices for thermoelectric power generation between 100 and 300 ℃. *Energ Environ Sci* **9**, 3120-3127 (2016).

9 Lu, X. *et al.* High-Efficiency Thermoelectric Power Generation Enabled by Homogeneous Incorporation of MXene in $(Bi,Sb)_2Te_3$ Matrix. *Advanced Energy Materials* **10**, 1902986 (2019).

10 Deng, R. *et al.* High thermoelectric performance in $Bi_{0.46}Sb_{1.54}Te_3$ nanostructured with ZnTe. *Energ Environ Sci* **11**, 1520-1535 (2018).

11 Hu, X., Nagase, K., Jood, P., Ohta, M. & Yamamoto, A. Power Generation Evaluated on a Bismuth Telluride Unicouple Module. *J Electron Mater* **44**, 1785-1790 (2015).

12 Jiang, Z. *et al.* Achieving High Thermoelectric Performance in p-Type BST/PbSe Nanocomposites through the Scattering Engineering Strategy. *ACS Applied Materials & Interfaces* **12**, 46181-46189 (2020).

13 Poudel, B. *et al.* High-Thermoelectric Performance of Nanostructured Bismuth Antimony Telluride Bulk Alloys. *Science* **320**, 634-638 (2008).

14 Hu, L. *et al.* Leveraging Deep Levels in Narrow Bandgap $Bi_{0.5}Sb_{1.5}Te_3$ for Record-High zT_{ave} Near Room Temperature. *Adv Funct Mater*, 2005202, (2020).

15 Qin, H. *et al.* Simultaneously Improved Thermoelectric and Mechanical Properties Driven by MgB2 Doping in $Bi_{0.4}Sb_{1.6}Te_3$ Based Alloys. *Advanced Electronic Materials* **7**, 2100173 (2021).

16 Yang, G. *et al.* Ultra-High Thermoelectric Performance in Bulk BiSbTe/Amorphous Boron Composites with Nano-Defect Architectures. *Advanced Energy Materials*, 2000757 (2020).

17 Jabar, B. *et al.* Enhanced power factor and thermoelectric performance for n-type $Bi_2Te_{2.7}Se_{0.3}$ based composites incorporated with 3D topological insulator nanoinclusions. *Nano Energy* **80**, 105512 (2021).

18 Zhai, R. *et al.* Enhancing Thermoelectric Performance of n-Type Hot Deformed Bismuth-Telluride-Based Solid Solutions by Nonstoichiometry-Mediated Intrinsic Point Defects. *ACS Appl Mater Interfaces* **9**, 28577-28585, (2017).

19 Xiong, C. *et al.* Achieving High Thermoelectric Performance of n-Type $Bi_2Te_{2.79}Se_{0.21}$ Sintered Materials by Hot-Stacked Deformation. *ACS Appl Mater Interfaces* **13**, 15429-15436 (2021).

20 Sun, Y. *et al.* Simultaneous Regulation of Electrical and Thermal Transport Properties of N-Type Bi2Te3 via Adding Excessive Te Followed by Se Doping. *ACS Applied Energy Materials* **4**, 4986-4992 (2021).

21 Liu, W.-S. *et al.* Thermoelectric Property Studies on Cu-Doped n-type $Cu_xBi_2Te_{2.7}Se_{0.3}$ Nanocomposites. *Advanced Energy Materials* **1**, 577-587, (2011).

22 Hao, F. *et al.* Enhanced Thermoelectric Performance in n-Type Bi_2Te_3-Based Alloys via Suppressing Intrinsic Excitation. *ACS Appl Mater Interfaces* **10**, 21372-21380 (2018).

23 Kuroki, T. *et al.* Thermoelectric Generation Using Waste Heat in Steel Works. *J Electron Mater* **43**, 2405-2410 (2014).

24 Company, K. L. *Thermoelectric device type: KTGM161-18*, <http://www.kelk.co.jp> (accessed April, 2021)

25 Technology.Inc, H.-z. *Thermoelectric device type: The HZ-2 watt module*, < http://www.hi-z.com> (accessed April, 2021)

26 Ying, P. *et al.* Towards tellurium-free thermoelectric modules for power generation from low-grade heat. *Nat Commun* **12**, 1121 (2021).

27 Liu, Z. *et al.* Demonstration of ultrahigh thermoelectric efficiency of ~7.3% in Mg_3Sb_2/MgAgSb module for low-temperature energy harvesting. *Joule* **5**, 1196-1208 (2021).

28 Wikipedia.org. *Abundance of elements in Earth's crust*, <www.encyclopedia.thefreedictionary.com> (accessed April, 2021)

29 *Shanghai metals market*, <www.metal.com/price> (accessed April, 2021)

30 *CBC Metal*, <www.cbcie.com/price> (accessed April, 2021)
