# Supplementary material for: A record thermoelectric efficiency in tellurium-free modules for low-grade waste heat recovery
Source: Nat Commun. 2022 Jan 11;13:237. doi: 10.1038/s41467-021-27916-y (PMC8752736; doi:10.1038/s41467-021-27916-y)
Supplement: Supplementary file 2 — Reporting Summary [file 41467_2021_27916_MOESM2_ESM.pdf]

Corresponding author(s): Yanzhong Pei

Lasing Reporting Summary

Nature Research wishes to improve the reproducibility of the work that we publish. This form is intended for publication with all accepted papers reporting claims of lasing and provides structure for consistency and transparency in reporting. Some list items might not apply to an individual manuscript, but all fields must be completed for clarity.

For further information on Nature Research policies, including our data availability policy, see Authors & Referees.

Experimental design

Please check: are the following details reported in the manuscript?

1. Threshold

   Plots of device output power versus pump power over a wide range of values indicating a clear threshold

   ☐ Yes ☒ No — Lasing experiment has not been performed in this work.

2. Linewidth narrowing

   Plots of spectral power density for the emission at pump powers below, around, and above the lasing threshold, indicating a clear linewidth narrowing at threshold

   ☐ Yes ☒ No — Lasing experiment has not been performed in this work.

   Resolution of the spectrometer used to make spectral measurements

   ☐ Yes ☒ No — Lasing experiment has not been performed in this work.

3. Coherent emission

   Measurements of the coherence and/or polarization of the emission

   ☐ Yes ☒ No — Lasing experiment has not been performed in this work.

4. Beam spatial profile

   Image and/or measurement of the spatial shape and profile of the emission, showing a well-defined beam above threshold

   ☐ Yes ☒ No — Lasing experiment has not been performed in this work.

5. Operating conditions

   Description of the laser and pumping conditions
   Continuous-wave, pulsed, temperature of operation

   ☐ Yes ☒ No — Lasing experiment has not been performed in this work.

   Threshold values provided as density values (e.g. W cm^{-2} or J cm^{-2}) taking into account the area of the device

   ☐ Yes ☒ No — Lasing experiment has not been performed in this work.

6. Alternative explanations

   Reasoning as to why alternative explanations have been ruled out as responsible for the emission characteristics
   e.g. amplified spontaneous, directional scattering; modification of fluorescence spectrum by the cavity

   ☐ Yes ☒ No — Lasing experiment has not been performed in this work.

7. Theoretical analysis

   Theoretical analysis that ensures that the experimental values measured are realistic and reasonable
   e.g. laser threshold, linewidth, cavity gain-loss, efficiency

   ☐ Yes ☒ No — Lasing experiment has not been performed in this work.

8. Statistics

   Number of devices fabricated and tested

   ☐ Yes ☒ No — Lasing experiment has not been performed in this work.

   Statistical analysis of the device performance and lifetime (time to failure)

   ☐ Yes ☒ No — Lasing experiment has not been performed in this work.
